# Dynamic Fluctuations Provide the Basis of a Conformational Switch Mechanism in Apo Cyclic AMP Receptor Protein

**DOI:** 10.1371/journal.pcbi.1003141

**Published:** 2013-07-18

**Authors:** Burcu Aykaç Fas, Yusuf Tutar, Türkan Haliloğlu

**Affiliations:** 1 Department of Chemical Engineering and Polymer Research Center, Boğaziçi University, Bebek, İstanbul, Turkey; 2 Department of Chemistry, Department of Biochemistry and CUTFAM Research Center, Faculty of Medicine, Cumhuriyet University, Sivas, Turkey; UNC Charlotte, United States of America

## Abstract

*Escherichia coli* cyclic AMP Receptor Protein (CRP) undergoes conformational changes with cAMP binding and allosterically promotes CRP to bind specifically to the DNA. In that, the structural and dynamic properties of apo CRP prior to cAMP binding are of interest for the comprehension of the activation mechanism. Here, the dynamics of apo CRP monomer/dimer and holo CRP dimer were studied by Molecular Dynamics (MD) simulations and Gaussian Network Model (GNM). The interplay of the inter-domain hinge with the cAMP and DNA binding domains are pre-disposed in the apo state as a conformational switch in the CRP's allosteric communication mechanism. The hinge at L134-D138 displaying intra- and inter-subunit coupled fluctuations with the cAMP and DNA binding domains leads to the emergence of stronger coupled fluctuations between the two domains and describes an *on state*. The flexible regions at K52-E58, P154/D155 and I175 maintain the dynamic coupling of the two domains. With a shift in the inter-domain hinge position towards the N terminus, nevertheless, the latter correlations between the domains loosen and become disordered; L134-D138 dynamically interacts only with the cAMP and DNA binding domains of its own subunit, and an *off state* is assumed. We present a mechanistic view on how the structural dynamic units are hierarchically built for the allosteric functional mechanism; from apo CRP monomer to apo-to-holo CRP dimers.

## Author Summary

Protein dynamics are central in allosteric communication. The cooperative character of atomic motions is key in the propagation of the allosteric signal and in the protein functioning. Here, we explored the dynamics of cAMP Receptor Protein (CRP) as an apo unbound monomer/dimer and a holo dimer by molecular dynamics simulations combined with the Gaussian network model analysis. The cAMP binding allosterically promotes CRP to bind specifically to the DNA. To this, our results show how the residue fluctuations provide the basis of an allosteric communication mechanism and describe a conformational switch with its *off* and *on states* and how this behavior is evolved from apo CRP monomer to apo-to-holo CRP dimers. The dynamic infrastructure needed for the coordination between the effector and DNA binding domains is largely observed in apo CRP monomer. This is an example of the pre-existence of functional dynamic states in the smaller subunits of the structure and the functional importance of the inter-domain hinges. The dynamics of the holo state pre-exist in the apo state, but disorganized in the way the cAMP and DNA binding sites interact. The cAMPs organize the couplings and elicit proper communication.

## Introduction

The *Escherichia coli* cAMP Receptor Protein (CRP) (also known as Catabolite Activator Protein, CAP) activates the transcription of more than 150 genes. Upon binding of cAMP (cyclic AMP), the transcriptional activity of CRP is altered resulting in a change in the affinity for its target CRP-dependent promoter region on the DNA. CRP is then able to recruit RNA polymerase (RNAp) for the transcription activation to begin.

CRP is a 47 kDa homodimer with 209 amino acid residues in each monomer where individual subunits fold into two domains [1]. The N-terminal domain, extending from V1 to N133, contains the primary cAMP binding site (*anti* cAMP) and mediates subunit-subunit interactions. This domain is formed by α helices A, B, C and eight β strands 1 to 8. The C-terminal domain extends from V139 to R209 with α helices D, E, F and four β strands 9 to 12, which has the helix–turn–helix (HTH) motif involved in the specific DNA and secondary cAMP binding (*syn* cAMP). Two domains are connected by the linker or hinge region L134-D138 [2]. The cAMP molecules in the primary cAMP binding sites interact mainly with residues G71, E72, R82, S83, T127 and S128 of the other subunit [3]. The active state that initiates transcription is accepted to be with two *anti* cAMPs bound form. The *syn* cAMP molecules can bind CRP only when the two *anti* cAMPs are bound. The *syn* cAMPs interact with the HTH motif (mainly R180) and β strand of the cAMP binding domain (E58), the DNA, and A135 of the other subunit [4]. Two cAMP molecules (*anti* and *syn*) bind to each subunit, making a total of four cAMPs per dimer. The binding of four cAMPs to CRP occurs at millimolar concentrations, yet the cAMP concentration is at micromolar levels *in vivo* [5]. The role of CRP-cAMP_4_ complex in the transcription mechanism is yet unknown [2].

**Figure 1:**
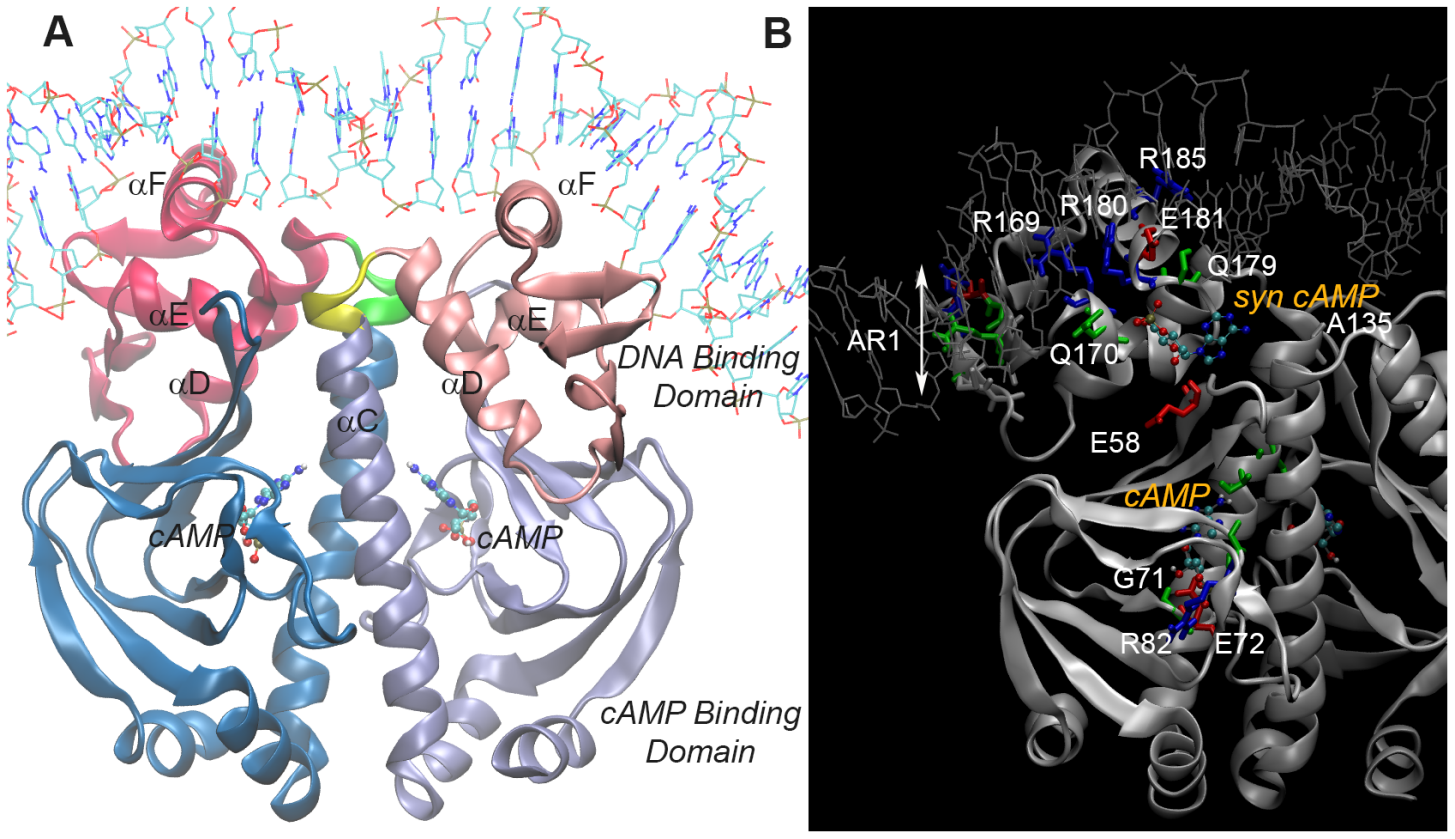
Holo CRP crystal structure and functional sites. ***A***. The CRP structure in complex with DNA and cAMP (PDB: 1CGP). The N-terminal cAMP binding domains (V1-N133), the C-terminal DNA binding domains (V139-R209) and the hinge regions (L134-D138) connecting the two domains in subunits A/B are represented in cyan/ice blue, dark pink/light pink and green/yellow, respectively. The α-helices C, D, E, F and bound cAMP molecules are indicated for both subunits. ***B***. The residues for the primary cAMP binding -G71, E72, R82, S83, T127, S128 (subunit B)-, the secondary cAMP binding –E58, A135, R180-, the specific DNA binding –R180, E181, R185-, the nonspecific DNA interactions –R169, Q170, S179, and the RNAp interaction –A156-Q164 (AR1) are labeled. The residues are colored white, blue, red and green corresponding to the residue types non-polar, basic, acidic and polar, respectively. The approximate position of the secondary cAMP is placed, considering the dimer CRP with four cAMPs (PDB: 2CGP). Figures are generated using VMD 1.8.7 [68].

CRP dimer interacts with DNA by a two-fold symmetric consensus DNA sequence. R180, E181 and R185 are the key residues of the HTH motif involved in specific protein-DNA interactions [6], whereas the nonspecific DNA binding residues are R169, Q170 and S179 [7] with the other DNA binding sites [8] D138, V139, T168, C178, T182, G184, K188, H199, and G200. Next to the HTH motif, residues A156-Q164 (activating region 1, AR1) are responsible for the transcription activation of *lac* class I and class II CRP-dependent promoters. The photo-cross-linking experiments [9] indicate that the C-terminal domain of the RNAp α-subunit (αCTD) binds one of the CRP subunits contacting AR1, whereas the other CRP subunit makes contacts with the other parts of the RNA polymerase. Then αCTD interacts with the minor groove adjacent to the DNA site for CRP. Thus, the transcription activation at *lac* promoter involves both protein-protein and protein-DNA interactions [10]–[13]. In class II promoters, the interaction with RNAp is complemented by two more activating regions of residues H19, H21, E96, and K101 (AR2) and K52–E55 and E58 (AR3) [14]. The X-ray crystal structure of holo CRP and the functional sites are shown in Figure 1A and B, respectively.

Within the sequence of events up to the transcription activation, the structural and dynamic characteristics of CRP and the activation mechanism through ligand binding can best be described by the comparison of the apo and holo states. The structures of holo CRP with and without the DNA have been elucidated since its first isolation from *E. Coli* in 1970 [15]. Several biochemical, genetic and biophysical experiments [2], [5], [13], [14], [16] along with computer simulations [17]–[19] have been performed to understand the cAMP allosteric switch mechanism from the inactive to active states. The three previous simulations [17]–[19] were based on the crystal structure of holo CRP dimer from which the apo state and the state with a single cAMP were also modeled. There are several recent studies and reviews addressing the allostery in CRP [2], [14], [19]. The NMR solution structure of apo CRP [20] and X-ray crystal structures of holo CRP [21], [22] have contributed significantly to the understanding of allosteric mechanism in play [20], [23].

The basic mechanism of allosteric control is the transmission of the signal from the cAMP to DNA binding domains upon *anti* cAMP binding and the interaction of cAMP adenine base atoms with the side chain hydroxyls of residues T127 and S128. This induces a coil-to-helix transition and the elongation of C-terminus of C-helix by 11 residues. The change in the secondary structure content with a rearrangement of the hinge [14], [23]–[25] directs the domain movements and triggers the rotation and translation of the DNA binding domain placing the F-helices into the required orientation to enter into the major grooves of the DNA. The shift in the inter-domain hinge was first observed by a structural inspection at the secondary structure level by NMR [24]. The hinge region appears to play a key role by modulating the inter-domain interactions and stabilizing the altered domain movements leading to the transcriptional activation [2], [14], [20]. The two states of the coiled-coil and the transition towards the ordered form coupled to the ligand binding functions as a regulatory switch. This ensures the precise allosteric control during the protein's functioning [20]. These studies suggest that the rearrangement of the hinge with the ligand binding is a critically conserved feature that controls the global allosteric transformation in CRP–family structures [14]. Thus, the majority of the CRP* (cAMP independent mutant of CRP) mutations in apo CRP are found to achieve proper allosteric transition by modifying the hinge-mediated inter-domain network pre-existing in apo CRP [14], [26], [27]. Although many X-ray crystal/NMR structures of different states of CRP are available, further structural information on the naturally occurring functional states of CRP, like CRP-cAMP_1_, CRP-cAMP-RNAp, is required for a complete understanding of CRP's allosteric mechanism and the functional implication of each state.

Further to the structural understanding of apo and holo states, allostery was also put forward as a dynamic relationship [28], [29]. Besides a mechanical view [30], [31] of structural changes, the dynamics is also of interest in the allosteric regulation of protein activity. With the changes in the protein motion, an allosteric communication may evolve [29]. Here, the dynamics of apo CRP monomer/dimer and holo CRP are explored computationally by extensive Molecular Dynamics (MD) simulations combined with the Gaussian Network Model (GNM) [32], [33] analysis of MD sampled conformations. The GNM analysis was also performed on apo CRP NMR solution structures and the holo CRP crystal structure. We mainly focus on how the fluctuation dynamics provide the basis for an allosteric communication and describe a conformational switch with its *off* and *on states* and how this behavior is evolved from the dynamics of apo CRP monomer to apo-to-holo CRP dimer. The dynamic infrastructure for the coordination between the effector and DNA binding domains is largely observed in apo CRP monomer. This is an example of the pre-existence of functional dynamic states in the smaller subunits of the structure and also the fact that the inter-domain hinges are not simply linkers that connect the two domains but coordinate the global structural motion, where the key dynamic states are pre-encoded [34].

## Results/Discussion

### Apo CRP as an unbound monomer and dimer

**Figure 2:**
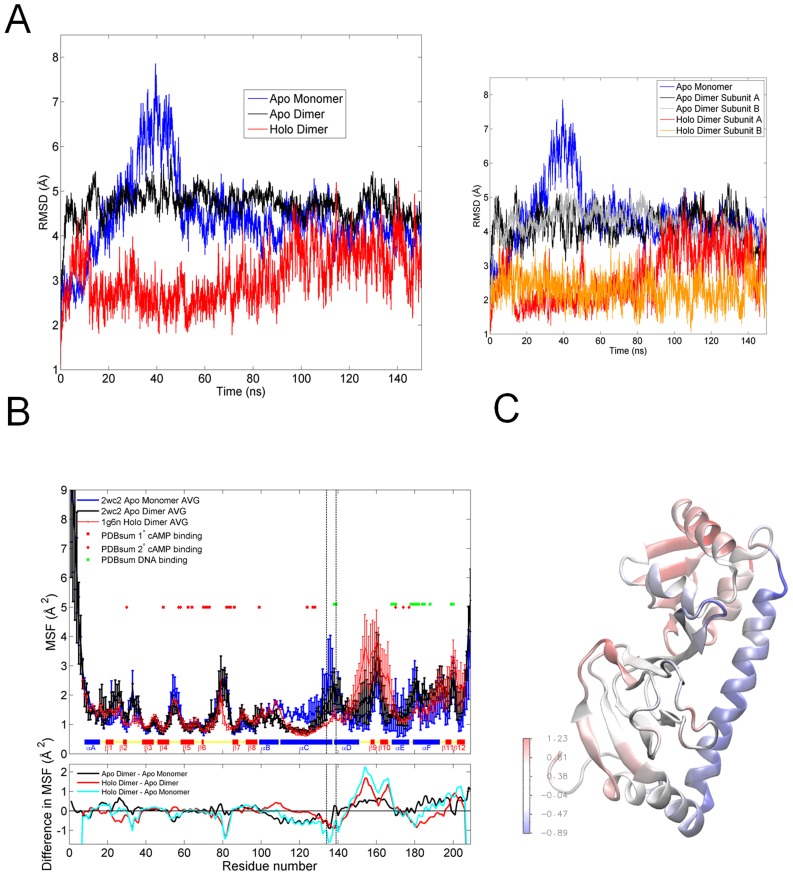
Dynamic fluctuations of apo CRP monomer/dimer and holo CRP by MD simulations. ***A.*** RMSD of the sampled conformations from the initial energy-minimized structure for apo CRP monomer/dimer and holo CRP along the 150 ns MD simulations. Comparisons between the RMSDs of different states are given in the inset figure. ***B.*** The MSF of residues for apo CRP monomer/dimer and holo CRP. The values are averaged over parallel runs for all states, and averaged over subunits for the dimers. The difference-MSF of different states is given as a sub-plot. ***C*** **.** The difference-MSF of apo CRP dimer (averaged over subunits) and apo CRP monomer.

The root-mean-square deviation (RMSD) profiles of the conformations sampled by the MD simulations of apo CRP monomer/dimer and holo CRP dimer are given Figure 2A. Apo CRP monomer and dimer reach similar RMSD values (∼5 Å) in a time window of 150 ns, yet the structural fluctuations are higher for the monomeric state in the first 50 ns. The parallel runs of apo CRP monomer display different profiles as a result of a kink in the C-helix at residues A121-R122 (Figure S2A). The rise of the RMSD in the second run is due to progression of the kink further along the simulation. The main structural change observed during the simulations for apo CRP dimer is the conformational change observed in the C-terminal of C- and F-helices of both subunits, which is more dramatic in apo CRP monomer. In apo CRP NMR structure, the V126-F136 region of C-helix is found to be coiled [20], whereas we have observed coil-helix transitions in all apo CRP monomer and dimer MD simulations (See Figure S1A, B for RMSD plots of all CRP MD simulations). The latter could be due to unstable and intrinsic conformational preferences of residues in the C terminal of C-helix, unless the Amber force field used over stabilizes α-helices [35], [36]. The holo CRP dimer, on the other hand, displays lower RMSD values with a conformationally stable C-helix. An average RMSD value of 3 Å is mainly due to the reorientation of the DNA binding domain of subunit A.

To this end, the MD simulations suggest that apo CRP monomer is relatively stable, although larger conformational space is accessible in this state. The apo dimer state is more stable and the holo state represents the most stable form. The biochemical studies indicated the role of cAMP binding in modulating the equilibrium between the monomer and dimer forms and this could be important for the different steps of the regulatory mechanism [37].

### Fluctuations and correlations by MD simulations

Figure 2B presents the mean-square fluctuations (MSF) (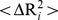) in residue positions for apo CRP unbound monomer/dimer and holo CRP dimer averaged over all MD runs with standard deviation values (See Figure S1B for MSF profiles as is for all seven runs). The differences between average MSF values of different states are given as a sub-plot. The difference-MSF of apo CRP monomer and dimer (averaged over subunits) is color-coded on a snapshot of apo CRP monomer in Figure 2C.

The MSF profiles display that the loop regions tend to have higher fluctuations, whereas β strands and α helices show more restricted motion as expected. Unbound monomer shows significantly higher fluctuations compared to apo/holo dimers for residues G33, D53-K57 (overlapping the RNAp interacting residues K52-E58 region/AR3), dimerization interface of C-helix (P110-T127), linker/hinge region (L134-D138), and α helices E and F (Q170-R180), which overlap with some of the secondary cAMP binding sites (Q170, Q174, G177, R180) and nonspecific (R169, Q170, S179)/specific (R180, E181, R185) DNA binding sites. Dimerization stabilizes the C-helix at the interface and decreases the mobility of the primary cAMP binding (T127 and S128) and DNA binding residues. Moreover, the binding of the cAMPs further stabilizes the inter-domain hinge and the DNA binding residues.

**Figure 3:**
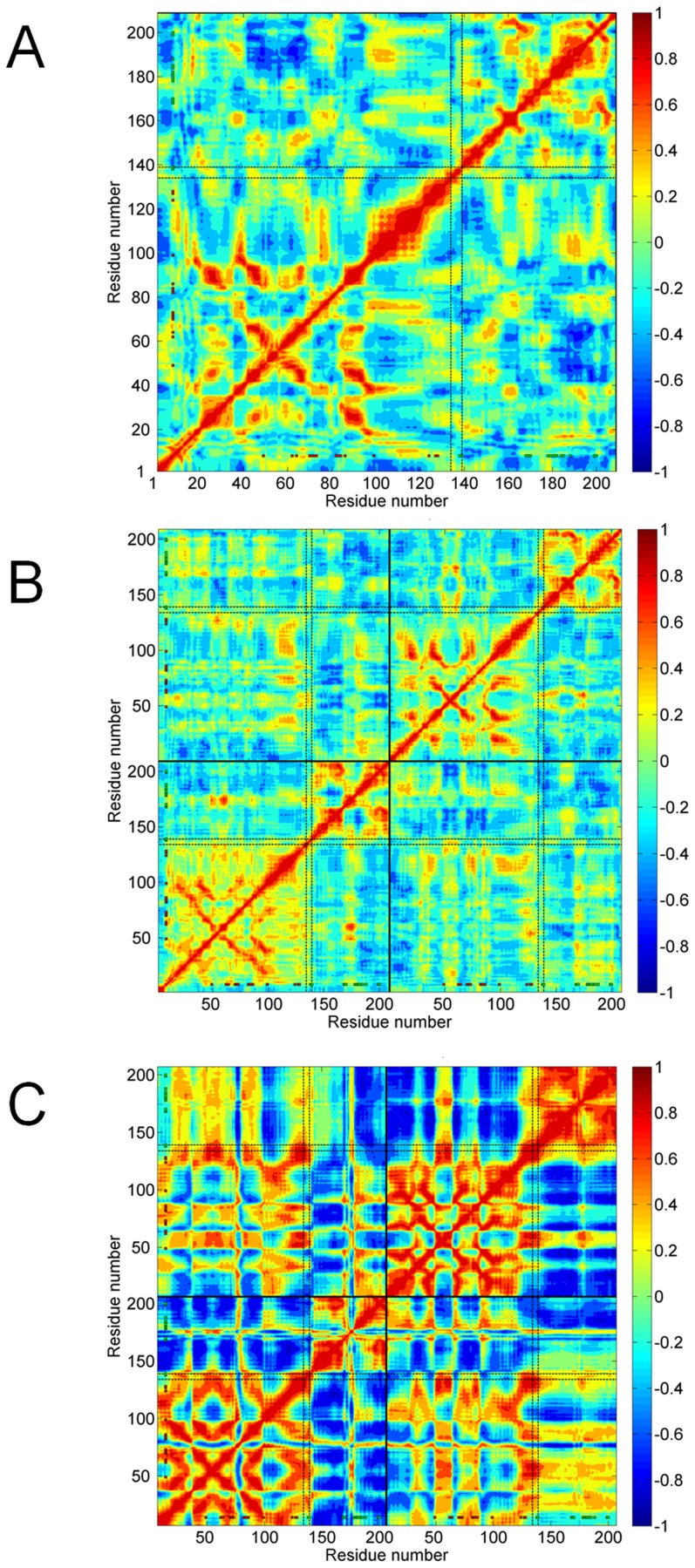
Dynamics network of apo CRP monomer/dimer and holo CRP by MD simulations. The correlation between residue fluctuations based on first ten essential modes is calculated as an average over three parallel MD runs for apo CRP monomer/dimer and holo CRP MD trajectories. The correlation maps are given for: (***A***) apo CRP monomer AVG, (***B***) apo CRP dimer AVG, and (***C***) holo CRP (single run).

The DNA binding and RNAp interacting sites, as well as the inter-domain hinge region, appear more stabilized with dimerization. CRP as a dimer should be in a more favorable dynamic state for the DNA and RNAp binding as well as for the secondary cAMP binding. Nevertheless, most of the primary cAMP binding sites' dynamics is already defined in the monomeric form. The experimental evidence suggests that the cAMP binds to the dimer [37] and the latter might still be taken as a hint of the dynamics for the order of events in various binding interactions of CRP. For example; the secondary cAMP binding follows the binding of the primary cAMPs [14]. The correlations between residue fluctuations are also of interest here to understand the cooperativity in residues' motion. Figures 3A–3C display the correlations between residue fluctuations based on the first ten essential modes for apo CRP monomer/dimer and holo CRP dimer, respectively.

#### Dynamic network in Apo CRP monomer and dimer

Correlations between residue fluctuations exist within the indiviual domains and between the domains. For the monomer form, within the cAMP binding domain, the most significant correlations that exist in the average correlation map (Figure 3A) are in the region R82-K100 and I20-I70, which includes several primary cAMP binding residues. Additionally, the fluctuations of residues L124, T127 and S128 are coupled to those of S62, L64 and I70 in the first two parallel runs, which are all primary cAMP binding sites. Yet, this coupling is not observed in the last run and thus appears weak on the average map (Figures S2A and Figure 3A). Additionally, K100-P110 region (B-helix) correlates strongly with different regions of the cAMP binding domain as can be seen from the maps of the individual runs (Figures S2A) that fades out in the average map (Figure 3A). K100-P110 region has a functional importance in CRP's interaction with cytidine repressor (CytR) [2]. P110S substitution was shown to perturb CRP/CytR cooperativity [38]. Within the DNA binding domain, mainly specific DNA binding sites communicate with the A156-Q164 region (AR1), which is one of the RNAp interacting sites. Regarding the inter-domain correlations, K100-P110 region (B-helix) in the cAMP binding domain correlates with residues around R185 in the DNA binding domain in the average map (Figure 3A). There are also other correlated regions in the correlations maps of the individual runs (Figure S2A), which become weaker in the average map. The main emphasize should be the larger conformation space and instable dynamic states of apo CRP monomer. These correlations will be further looked in with the GNM cross-correlations of the mainly sampled conformers.

The dynamic domains that are defined mainly by the cAMP and DNA binding domains are more apparent with dimerization. For the dimer case, the cAMP and DNA binding domains appear as quasi- independent dynamic units with the inter-domain hinge L134-D138 being more stabilized in comparison to apo CRP monomer (Figure 3B). Within the cAMP and DNA binding domains, the residues identified in the average and individual correlation maps of the unbound monomer, could also be observed in apo CRP dimer. The correlated residue fluctuations become stronger within the DNA binding domain. Further, sub dynamic domains within the DNA binding domain emerges with a hinge site at G173-V176 and an additional flexible segment at P154-A156 (Figure S2B, map of the first run). G173-V176 that connects two segments of the DNA binding residues correlates with L50-T90, which overlaps with most of the primary cAMP binding sites in the average map (Figure 3B) and the individual map of the first/longest run (Figure S2B). Here, the L134-D138 hinge coordinates the two subunits of apo CRP dimer, while interacting with G173-V176 in the DNA binding domain and K52-E58 in the cAMP binding domain. Moreover, although it is not as clear in the average map, the individual maps display that the two domains interacts also through the P154-A156 site in the DNA binding domain. The intra-/inter-domain and inter-subunit correlations become stronger with the holo state.

### Fluctuations and correlations by Gaussian Network Model analysis

**Figure 4:**
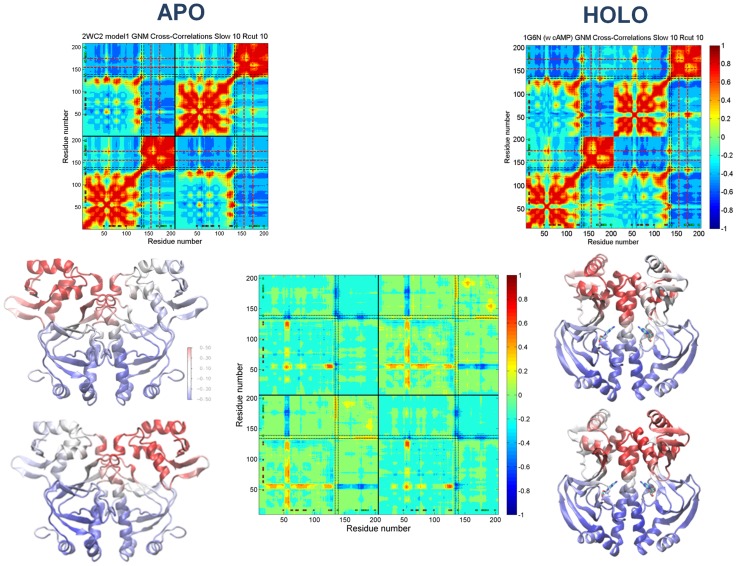
GNM cross-correlations of apo/holo CRP and the switch behavior. Comparison of the correlations between residue fluctuations in average ten slowest modes for the apo CRP NMR solution structure (PDB: 2WC2, model 1) and holo CRP X-ray crystal structure with the two cAMPs bound (PDB: 1G6N). Below, the ribbon diagrams color coded with the correlation values of the L134-D138 hinge (average) with the rest of the structure are given. The difference-correlation map of the two correlation maps is given in the middle.

The correlations between residue fluctuations are further evaluated below through the GNM analysis of the MD sampled conformational states of apo CRP monomer/dimer and holo CRP (Figures S4A–G). Apo NMR model and holo crystal structures are also analyzed by GNM (Figures S5, Figure 4). The hinge sites are the key mechanical sites for the cooperative motion that we observe in the correlation maps. Although apparent from the MD mean-square fluctuations (Figure 2B), the GNM predictions by the slow mode shapes of MD cluster best members clearly reveals the most global hinge region of CRP as the inter-domain hinge L134-D138 (Figure S7). The intra-domain hinges/flexible sites K52-E58 of the cAMP binding domain and G173-V176 of the DNA binding domain are evident in apo and holo CRP dimers, which are also observed in apo CRP monomer yet with less stability. On the other hand, the intra-domain flexible site P154-A156 of the DNA binding domain appears in apo CRP monomer and dimer, and becomes less evident in holo CRP dimer (See Figure S7F for the sites labeled on structure). This may imply that this site close to the RNAp binding site may have a plausible role in the cAMP unbound state.

#### Dynamic network in Apo CRP NMR and holo CRP crystal structures

Figure 4 displays the cross-correlation maps based on the average ten slowest modes and the ribbon diagrams color coded with the correlation values of the L134-D138 hinge of subunits A and B, with all other residues in a selected model of the apo CRP NMR solution structures (PDB: 2WC2, model 1) [20] and in the holo CRP X-ray crystal structure with the two cAMPs bound (PDB: 1G6N) [22]. A major difference in the internal dynamics between the apo and holo states is related to the dynamic behavior of the inter-domain hinge L134-D138, K52-E58 of the cAMP binding domain, and G173-V176 of the DNA binding domain. L134-D138, while being correlated with only the cAMP and DNA binding domains of its own subunit in the former, correlates with the cAMP and DNA binding domains of both subunits in the latter. The same behavior is observed in the majority of apo NMR model structures. A holo-like correlation dynamics is observed only in some NMR models (Models 5 and 10; See Figure S5A for all apo NMR models) as a weak population in the apo state ensemble.

The L134-D138 hinge coordinates the relative motion of the two domains and both CRP subunits in the holo state. There is yet a shift in the boundaries of the two domains, when L134-D138 becomes a part of the dynamic DNA binding domain in the apo state. L134-D138 loses its global hinge behavior and thus the coordinated motion of the domains and subunits weakens. This is observed with the weakening of the interactions between L134-D138 hinges of both subunits and between K52-E58 of the cAMP binding domain and the DNA binding domain (particularly with G173-V176). The latter is clearly observed with the difference-correlation values of the average correlation map of all NMR models and the holo crystal structure color-coded (Figure 4). The relatively high positive values in the DNA binding domains indicate that the L134-D138 hinges display higher correlation values with the DNA binding domains of their own subunits in the apo state. The relative more negative values in the L134-D138 hinge, K52-E58 of the cAMP binding domain, and G173-V176 of the DNA binding domain of the other subunit show that the L134-D138 hinge displays stronger correlations with the latter regions in the holo state. Along, the relatively high negative values between K52-E58 and G173-V176 (and whole DNA binding domain) mean that the two domains are coupled stronger in the holo state or vice versa for the apo state.

A very similar difference-correlation pattern is observed if the average cross-correlation map of the holo MD cluster best members is used in place of the holo crystal structure. All holo conformations show the same correlation behavior, including the two conformations from a previous holo CRP MD simulation study [18].

#### Dynamic network in MD sampled conformations

GNM predicts residue fluctuations and correlations around a given minimum conformational energy state. As a complementary analysis of the direct analysis of the MD trajectories, GNM can expand the MD sampled space through the analysis of mainly MD sampled conformations. To this, the conformations visited through the MD simulations of apo CRP monomer/dimer and holo CRP dimer were clustered for some favorable conformational states and then the cluster best members were analyzed by GNM. There appear 13, 6, 5, 8, 7, 6 and 3 clusters with a cluster radius of 3.0 Å; and 8, 4, 4, 4, 4, 3 and 2 clusters with a cluster radius of 3.5 Å, for three parallel runs of apo CRP monomer/dimer and a single run for holo CRP dimer, respectively. The number of clusters becomes considerably lesser and the accessible conformational space is more restricted with dimerization and further with the cAMP binding. The main conformational differences between different cluster best members of apo CRP monomer are the small fluctuations –one or two turns– in the length and orientation of the D, E and F helices of the DNA binding domain and in the length of the C-terminus of C-helix. The appearance of a kink near residues A121 and R122 of the C-helix also leads to some conformational differences with the formation of a salt-bridge between D68 and R123 in two parallel runs of apo CRP monomer. When R123 makes a salt-bridge with E72 (first monomer run), no kink appears in the C-helix. The role of R123 in stabilizing apo CRP by making salt interactions either by D68 or E72 was experimentally questioned and reported that D68-R123 is the salt-bridge responsible for the stabilization [39].

**Figure 5:**
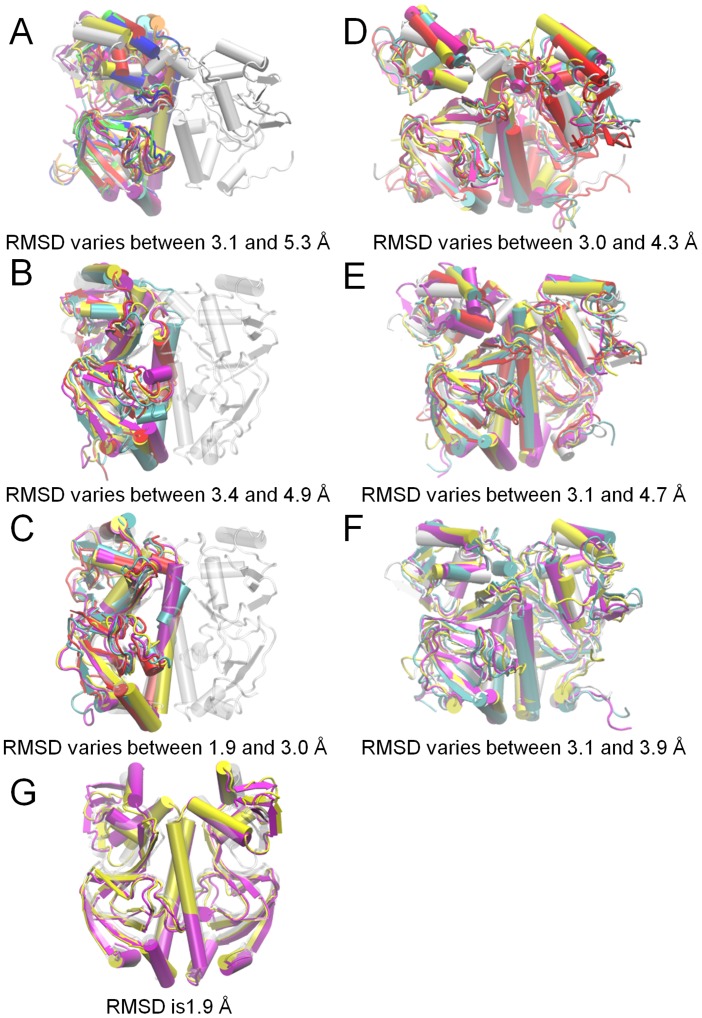
The alignment of MD cluster best members of apo CRP monomer/dimer and holo CRP. The superimposed MD best member structures of the clusters generated from three apo CRP monomer runs (***A, B, C***), three apo CRP dimer runs (***D, E, F***) and a holo CRP run (***G***) with a cluster radius of 3.5 Å. Colors of the structures match the colors of the clusters given in RMSD plots of cluster evolution in time (Figure S3). For example, the structure in cyan is the cluster best member no. 3, which is also colored in cyan. The white structures are the starting conformations for each run.

In apo CRP dimer, the differences are mainly in D- and F-helices of the DNA binding domain and the length of the C-terminus of C-helix of both subunits (Figure 5D–F). We observe both salt-bridges D68- R123 and E72- R123 interchangeably in apo CRP dimer simulations, yet only D68-R123/E72-R123 salt-bridge appears in apo NMR models/holo structures, respectively. The holo structure stays intact with an average RMSD value of 3 Å for the whole trajectory, except the reorientation of the DNA binding domain of subunit A to an open conformational state (Figure 5G). This can be explained by the disappearance of the crystal packing effects in the starting conformation with asymmetrically oriented DNA binding domains. This behavior was also reported in a previous simulation study [18]. The opposite behavior was yet observed in another earlier simulation study [17]. The unraveling of the DNA binding domain was observed in more recent MD simulations [19] performed for the three states of CRP (apo, single cAMP, two cAMPs) and the CRP/DNA complex, which is not observed in the present apo simulations.

Figures S4A–C display the GNM correlation maps of equilibrium fluctuations in the average ten slowest modes for the cluster best members of three apo CRP monomer runs. In Figure S4A, the correlations between residue fluctuations identify the cAMP and DNA binding domains with the inter-domain hinge fluctuating from position L134-D138 to N terminus and back in all cluster best members of 150 ns MD simulation. In the first cross-correlation map of Figure S4A, the strongest inter-domain correlations are between A48-Y63 and Q174-G177, which is located on the short helix αE of helix-turn-helix motif. Q174-G177 manages to display lesser correlations with the residue fluctuations of its own domain and associate with the cAMP binding domain through A48-Y63, which includes some of the primary (S62, L64) and secondary (K57, E58) cAMP interacting residues and RNAp interacting residues (AR3, K52-E58). Another region of the dynamic interaction between the DNA binding and cAMP binding domains are through residues P154/D155 and K22-K26/V43-V47/Y63-N65/A88-C92. The communication of the two domains gets weaker in the next four clusters and gets stronger again in the last three, suggesting that the cAMP and DNA binding domains interact transiently. The L134-D138 hinge is observed to interact with both K52-E58 of the cAMP binding domain and Q174-G177 of the DNA binding domain. Although the interaction of L134-D138 with Q174-G177 is conserved among the clusters, the strength of the correlation varies from one cluster to another. To this end, the C terminus of C helix displays transitions between helix and coiled states. Similar dynamic correlation patterns are observed in the parallel runs (Figure S4B and C), except a stronger correlation of V49 with the residues of C terminus of C helix and Q174-G177.

Figures S4D–F displays correlations between residue fluctuations in the average ten slowest modes for the cluster best members of the three apo CRP dimer runs. The residue fluctuations provide how cAMP and DNA binding domains form dynamic units and their cooperative motion is maintained by the inter-domain hinge L134-D138 and the regions K52-E58 of cAMP binding domain and P154-A156 and G173-V176 of DNA binding domain. Although, the main features of the dynamic patterns are conserved among the clusters, the strength of the correlations differs between L134-D138 and the cAMP and DNA binding domains. For apo CRP dimer (first run, Figure S4D), when we compare the two subunits in each map, L134-D138 displays a global hinge behavior in subunit A, whereas it becomes part of the DNA binding domain with a shift in its position towards N terminus in subunit B. The average correlation values of L134-D138 with the rest of the structure are color coded on the ribbon diagrams. L134-D138 display both intra-/inter-domain and inter-subunit correlations in subunit A and mainly intra-domain interactions in subunit B. These are coupled with the conformational preferences of the C-termini of C helices (more helical and coiled) and the correlations between the residue fluctuations of the cAMP and DNA binding domains (more ordered and disorganized), respectively. On the other hand, the dynamic correlations of the best members of the parallel MD simulations show that both subunits can behave as subunit A (or B) or subunits can switch with respect to the dynamic behavior of L134-D138 (See Figure S4E and S4F). The correlation maps of holo CRP dimer cluster best members show that the inter-domain hinge L134-D138 correlates with the cAMP and DNA binding domains of both subunits and there is a strong coupling between K52-E58 and G173-V176, which is almost the same behavior observed with the holo crystal structure (Figure S4G, Figure 4).

**Figure 6:**
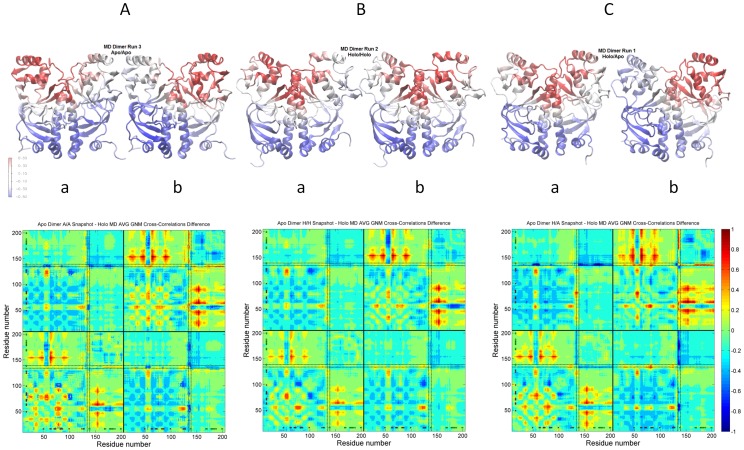
Ensemble of CRP conformations. Ensemble of structures (dynamic states) are selected according to their dynamic behavior among the MD cluster best member structures with a cluster radius of 3.5 Å. The ribbon diagrams are color coded with the correlation values of the L134-D138 region (average) with the rest of the structure. The difference-correlations maps for each structure in the ensemble with respect to the holo CRP dimer are given: (**A**) The L134-D138 hinge of both subunits (A/a& B/b) displays apo-like behavior with relatively strong coupling with the DNA binding domain of its own subunit (higher positive values), relatively weak coupling with the L134-D138, K52-E58 and G173-V176 regions of the other subunit (higher negative values), and weak coupling between the K52-E58 and G173-V176 sites (higher negative values). (**B**) This structure is the one that displays the closest dynamic behavior in overall to the holo state with respect the both subunits (A/a&B/b); theL134-D138 hinge is not strongly correlated with the DNA binding domain of its own subunit (lower positive values), and not weakly correlated with the K52-E58 and G173-V176 sites (lower negative values) compared to (**A**). (**C**) Subunit A/a displays more holo-like behavior while subunit B/b assumes apo-like behavior following the L134-D138 hinge dynamics. See (B) for the dynamics of subunit A/a and see (A) for the dynamics of subunit B/b.

Figure 6 displays an ensemble of dynamic behavior based on the selected MD cluster best members of apo CRP dimer. This ensemble of structures is color-coded on the ribbon diagrams with respect to the correlation values of the inter-domain hinge L134-D138 with all other residues. In each figure pair, left and right corresponds to values with respect to subunits A and B, respectively. In terms of the inter-subunit communication of the two DNA binding domains and the helicity content of the C termini of the C helices, the pairs are categorized as apo/apo, holo/holo and holo/apo, demonstrating the pre-existence of different states in the apo state in the absence of the ligand. The corresponding difference-correlations maps for each structure in the ensemble with respect to the holo CRP dimer are also given (Figure 6). Here, if the interaction of the L134-D138 hinge region with the K52-E58 and G173-V176 regions is closely examined, the difference-correlation maps help follow the dynamic behavior for the states of the apo structures with respect to the holo state (See Figure 6 legend). In all cases, the coupling between K52-E58 and G173-V176 in none of the apo structures (and states) is as strong as in the holo state. Interestingly, the P154-A156 region's dynamics is emphasized in all apo structures (monomer/dimer MD and NMR). Thus, for all three structures in the ensemble, there is a stronger correlation between the cAMP and DNA binding domains through P154-A156 compared to the holo state dynamics (See section *Dynamic network in apo CRP NMR and holo CRP crystal structures* for a more elaborated description of the difference-correlation maps).

### The inter-domain hinge as a means of allosteric switch in CRP

The GNM cross-correlation maps of apo NMR structures, holo CRP crystal structure and the conformations from the apo CRP monomer/dimer and holo CRP dimer MD simulations suggest a conformational switch mechanism mediated by the L134-D138 hinge in the allosteric communication of the DNA and cAMP binding domains. The network of cooperative fluctuations describes the *off* and *on states* of this switch: In the *on state*, the L134-D138 hinge displaying stronger fluctuations with the L134-D138 hinge of the neighboring subunit and the K52-E58 and G173-V176 regions in the cAMP and DNA binding domains, respectively, of both subunits, is able to coordinate the movement of the domains and the interactions in between. The coordinated behavior of the two DNA binding domains should provide the DNA binding residues with a more favorable dynamics for the DNA binding. On the other hand, the L134-D138 hinge is no longer a global hinge and only correlated with the DNA binding residues of its own subunit in the *off state*, when the coordination between the key functional elements of the structure is weakened. The *on state* and the *off state* are as well strongly coupled with the conformational preference of the C terminus of C helix. This preference is apparently correlated with the position of K52-E58 with respect to C helix.

The highly evolutionary conserved residues of the inter-domain hinge L134 (8), A135 (9), F136 (9), L137 (5), D138 (9) (ConSurf [40] scores are listed in parentheses; 9 to 1 referring to most conserved to most variable) should imply their certain conformational preferences and further support the functional importance of this linker. Similarly, L52-E58, P154-A156 and G173-V176 appear also as highly conserved (K52 (7), D53 (7), G56 (5), E58 (8); P154 (9), D155 (7), A156 (9) and G173 (7), Q174 (8), I175 (7), V176 (9)). The conserved nature of P154-A156 shows the dynamic importance of also this flexible site in the DNA binding domain and its communication with the cAMP binding domain. The overall evolutionary conservation profile of CRP (See Figure S6) demonstrates that the most conserved patches predominantly cover the latter sites. A large amount of mutational studies [14] have focused on the cAMP independent CRP variants (CRP*), often found to be mutated in the proximity of the inter-domain hinge region (T127/S128, D138, T140, G141, R142, A144). Other known mutation sites are D53, S62, Y99, H159, K52/H159, and L195. The major contributions of mutational studies could be achieved by considering the effects on the internal dynamics, in addition to interpretations solely based on the structural data.

As noted in the literature, upon binding of cAMP W85 is expelled into solvent and β4–β5 flap (K52-E58) moves towards C helix, resulting in hydrophobic interactions of I51, K57, M59, L61 with F136 [20]. The β4–β5 flap interacts with F136 of the hinge upon cAMP binding and stabilizes the L134-D138 inter-domain hinge. This, in other words, locks the *on state* with the coupled fluctuations of K52-E58 with the L134-D138, which in return reassumes its global hinge behavior for the inter-domain and then inter-subunit interactions; i.e., shifts the hinge region towards C terminus. The latter refers to the two end states of the allosteric transition pathway between apo and holo structures. Nevertheless, the GNM analysis of both NMR model structures and mainly MD sampled conformations shows that the dynamics of conformations may suggest that both states, yet the *on state* and the *off state* with respect to both subunits is rare within the time window of simulations.

The dynamic infrastructure for the allosteric communication pre-exists in the apo state, yet the fluctuations are not fully organized for a proper communication of the two domains and the two subunits. Binding of the cAMPs organizes the couplings and elicit proper communication. When we look at the dynamics of the unbound monomer, the elements of a plausible allosteric mechanism is still observed: The fluctuations of the position of the L134-D138 hinge to N terminus, the unstable correlated fluctuations of L134-D138 with K52-E58, and unstable correlated fluctuations between the DNA binding (P154-A156 and G173-V176) and cAMP binding domains. To this end, it is plausible to state the key dynamic elements of the allosteric functional mechanism is hierarchically built up but yet stabilized to the fully functional state with the association of the structural units; the dimerization and cAMP binding.

### MD simulations and GNM for internal fluctuations and allostery

GNM assumes an ensemble of conformations around a given protein structure topology and predicts residue fluctuations. GNM may expand the MD sampled space through the predictions on mainly MD sampled conformations. Clustering is one way to reduce the MD conformational space into a subset of conformational states, where the conformational ensemble could be enlarged from each. The cluster best members could be considered as some energetically favorable conformational states. As alternative to distance based metric such as clustering and PCA, GNM was previously used to characterize different conformational states and dynamics along MD trajectories [41]. We have also used this idea of plotting the frequency distributions of the eigenvalue of the first (or first few) GNM eigenvector of a series of MD snapshots to characterize different conformational states visited during the simulations. The frequency distributions were seen to be sensitive to the states of CRP. The frequency values for the cluster best members was distributed homogeneously over the frequency values of all conformations (data not shown), which also shows that the cluster best members could capture possible differences in the topology and dynamics of CRP.

The general patterns of the correlation maps are captured by the predicted correlations. Nevertheless, it is observed that, the larger the number of clusters for the MD conformations, the greater the difference between the MD/GNM cross-correlations of MD cluster best members. The differences here observed largest for the monomer (Figures S2A versus S4A–C) and the least for the holo structure (Figures 3C versus S4G) where the accessible conformational space is increasingly decreased. When there are large conformational changes, given the GNM calculations performed on mainly sampled conformations, the expansion in the conformational space could be larger compared to the relatively constrained cases. Also, for the MD cross correlations, averaging over whole trajectory might hide the dynamic behavior of less dominating conformational states.

GNM helps elaborating the dynamics assumed in each of these conformational states. Harmonic motion assumption and non-specificity in the underlying potential function are GNM's limitations together with the dependency on the given structure. On the other hand, PCA-based analyses of MD trajectories provide the dominant motion suggested by the MD time window, but it may change from one sampling window to another [42], [43]. Although usefulness of PCA analysis on insufficiently sampled MD trajectories may still be enhanced through multiple MD trajectories [44], the dominant dynamic behavior might have contributions from several conformational states through the trajectory and may not uncover the individual states well. The identified conformational states structurally might look similar, yet the dynamics can be affected with differences in some contacts if involving some key mechanical sites. The cooperative residue fluctuations may allow the propagation of the allosteric signal with the minimal structural changes in the mean conformation [45]–[48]. CRP variants, although having structurally poised DNA interfaces, was seen to bind to DNA with different binding affinities; whereas the S62F CRP mutant, although the DNA binding domain is not reoriented to the active conformation, could show strong DNA binding affinity [28], [29], [47]. This suggests that binding may entirely be driven by the conformational entropy change. To this end, the dynamic analysis here demonstrates that the apo state has predisposed dynamics for both *on* and *off state* of the allosteric switch mechanism without undergoing the major conformational changes.

It has previously been demonstrated that the sequence evolution correlates with structural dynamics [49], [50]. It is expected that the key residues that mediate cooperative fluctuations could be conserved or assume correlated mutations. These are basically hinge sites that have been also suggested to overlap the regions of maximum frustrations that have a role in the emergence of allosteric interactions [51]. Allosteric functional motion and the cooperative modes are closely related. The robustness of the low frequency cooperative modes should depend on their nearly invariant nature, placing their foundations on the core network of residues responsible for transmitting signals as suggested by [52]. The local perturbations could be coupled to these modes which possibly transmit the signal by inducing conformational and/or dynamic changes encoded in the structure's topology [42], [53]. Here, as a contribution to the understanding of the allosteric mechanism, we used GNM and MD simulations combined to suggest the dynamic infrastructure of a possible conformational switch mechanism from apo CRP monomer to dimer, then to holo CRP dimer. The key features of the allosteric dynamics are encoded in apo CRP dimer as well as in apo CRP monomer, providing a basis to elicit the transmission of a signal from the cAMP binding site to DNA binding domains. The use of MD sampled conformations along GNM allows having more than one conformational state to be used in the GNM analysis, which could particularly be important for the cases where there are several conformational states accessible with some topological differences.

### Conclusions

The MD simulations coupled with the GNM analysis has provided a mechanistic view on how the structural units are dynamically built up for a plausible allosteric functional mechanism; from apo CRP monomer to apo-to-holo CRP dimers. The dimerization restricts the conformational states accessible to the structure, so does the cAMP binding, towards a favorable dynamics for the DNA binding. The key dynamic elements; the inter-domain hinge L134-D138 and the K52-E58, P154-A156 and G173-V176 sites, provide the dynamic infrastructure starting from the monomeric state and the orchestration of which leads to the allosteric communication between the cAMP and DNA binding sites/domains. A switch mechanism appears with the main role of the global hinge L134-D138; the *on* and *off states* are evidenced in apo CRP dimer with the precursor dynamics as well observed in the monomeric form.

## Materials and Methods

### Molecular dynamics (MD) simulations

The MD simulations were performed for the dimer (subunits A and B) and the unbound monomer (subunit A) of apo CRP for a simulation time of 150 ns each with the initial structure of apo CRP NMR solution structure (PDB: 2WC2, model 11) [20], as well as a 150 ns holo CRP dimer run with the initial holo CRP X-ray crystal structure (PDB: 1G6N) [22]. Two parallel runs of 75 ns were performed for each apo CRP monomer and dimer with different initial structures (PDB: 2WC2, models 2 and 10) [20]. The details of the simulated systems are given in Table 1.

**Table 1:** Details of the simulated systems.

Model	No. of amino acids	Box dimension (Å)	No. of water molecules	Total no. of atoms	No. of Cl^−^ ions	Simulation time (ns)
Apo CRP monomer (1)	209	80.66	32,292	35,650	7	150
Apo CRP monomer (2)	209	73.72	27,519	30,877	7	75
Apo CRP monomer (3)	209	73.64	27,486	30,844	7	75
Apo CRP dimer (1)	418	89.18	48,072	54,788	14	150
Apo CRP dimer (2)	418	93.05	48,183	54,899	14	75
Apo CRP dimer (3)	418	90.42	43,728	50,444	14	75
Holo CRP dimer	401	82.51	43,578	50,069	2	150

*The truncated octahedron periodic boundary conditions were applied to each system.*

The Amber 8.0 [54], [55] and Amber 11 [56] biomolecular simulation programs were used in the MD simulations. The Amber ff03 [57] force field parameter set was used for the proteins/ions. Each system was solvated using TIP3P [58] water molecules in an octahedron periodic box. Histidine residues 17, 19, 21, 31, 159 and 199 were protonated for the states predicted by H^++^ server [59]–[61]. Initially, energy minimization was performed using 50 cycles of steepest descent algorithm, followed by the conjugate gradient method. Initial velocities were selected at random from the Maxwell-Boltzmann distribution at a temperature of 10 K, which was gradually increased to 300 K. The Berendsen thermostat and barostat [62] was used for the NPT ensemble (T = 300 K, P = 1 bar) with a time step of 2 fs. The SHAKE algorithm [63] was used as the bond constraints for all bonds involving hydrogens to eliminate the high frequency bond vibrations. A cutoff distance of 9 Å was used for the nonbonded interactions. The long-range interactions in electrostatic terms were corrected using the particle mesh Ewald method [64]. The equilibration periods were taken as 5 ns and 50 ns for the dimer and monomer trajectories, respectively.

### The simulation analyses

The MD trajectories were analyzed for the structural and dynamic properties, such as the Root-Mean-Square Deviation of MD sampled conformations from the initial structure, Mean-Square Fluctuations of residues and Cross-Correlation of Residue Fluctuations, where all the calculations are based on Cα atoms and performed by *ptraj* of AMBER toolset 1.5 [65]. The first ten essential modes are extracted from the MD trajectories using singular value decomposition of the fluctuation residue matrix [66]. The clustering of conformations was performed to reduce the sampled conformational space for relatively major conformational states. The MD conformations of dimer and unbound monomer CRP (saved every 4 ps) were clustered with the k-means method (*kclust* script) implemented in the MMTSB toolset [65]. In clustering, cluster radii of 2.0, 2.5, 2.7, 3.0 and 3.5 Å was used. Smaller cluster radii values produce more clusters, which are often unmanageable and insignificantly different from each other. The best members (cluster centroids/the nearest conformation to the centroid) of clusters with 3.5 Å were chosen for the analysis. The cluster populations are reported in Table 2 for the cluster radius of 3.5 Å. Cluster best members were further analyzed by using the Gaussian Network Model.

**Table 2:** Cluster populations.

	Cluster Populations for a Cluster Radius of 3.5 Å								
Structure/Cluster #	1	2	3	4	5	6	7	8	Total
Apo CRP monomer (1)	3,355	4,500	2,631	13,712	7,670	1,968	1,785	1,879	37,500
Apo CRP monomer (2)	3,040	13,231	702	1,777					18,750
Apo CRP monomer (3)	11,247	6,658	700	145					18,750
Apo CRP dimer (1)	31,567	4,324	1,334	275					37,500
Apo CRP dimer (2)	5,860	11,581	1,059	250					18,750
Apo CRP dimer (3)	11,689	6,192	869						18,750
Holo CRP dimer	22,461	15,039							37,500

### The GNM analysis

The Gaussian Network Model (GNM) [32], [33] is a one dimensional elastic network (EN) model. GNM assumes that the residues undergo Gaussian fluctuations about their equilibrium positions, where the residues represented by their backbone alpha carbon atoms (Cα) interact if they are within a certain cut-off distance. The interactions between all residue pairs in the network are represented by the Kirchhoff (or connectivity) matrix **Γ**. The correlation between the residue fluctuations of Δ**R_i_** and Δ**R**j is given in (Eq. 1) [33].
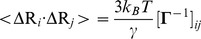
(1)where k_B_ is the Boltzmann constant, T is the absolute temperature in degrees Kelvin and γ is the force constant of the elastic potential function. The correlation matrix in Eq. 1 can also be expressed as the linear superimposition of N-1 eigenmodes as
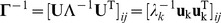
(2)
**U** is the matrix of eigenvectors **u**_k_, where k refers to the kth eigenvector that gives the displacements of the residues along the kth mode. The kth eigenvalue, λ_k_, is proportional to the frequency of the kth mode of motion. The normalized cross-correlation values of residue fluctuations vary in the range [−1, 1], referring to the limits of the negatively correlated 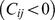 and positively correlated 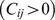 pairs in their fluctuations, respectively. The N-1 nonzero modes are obtained by the decomposition of the fluctuations of N residues of a structure, where the most cooperative/global modes are the first few modes called the slow modes. The square of these eigenvectors describes the mean square fluctuations of residues from equilibrium positions along the selected modes. The minima of the slow mode shapes describe the hinge regions that coordinate the cooperative motion of the structure in the given mode, which are usually responsible for the correlated movements of the domains [32], [33]. On the other hand, the hinge regions in the slowest individual modes are the sites where the sense of the residue correlations changes, the global hinge centers are located at the crossover between the positive and negative displacements [67].

Here, the GNM was used in combination with the other MD trajectory analysis approaches. A cut-off radius of 10 Å is used for all GNM calculations. The equilibrium residue fluctuations and cross-correlations of the cluster best members of each trajectory were calculated by the GNM. Additionally, the GNM analysis was also carried out for apo CRP NMR solution structures (PDB: 2WC2) [20], holo CRP X-ray crystal structures with DNA (PDB: 1CGP) [21] and without DNA (PDB: 1G6N) [22], and for the two conformations from a previous MD simulation study of holo CRP (PDB: 1G6N) [18], [22]. The GNM characterizes fluctuations of the molecule near an equilibrium state given by the molecule's Cα atomic coordinates. It thus expands the MD sampled space through the mainly sampled MD conformations, the cluster best members.

## Supporting Information

Figure S1 **Fluctuations of apo CRP monomer/dimer and holo CRP by MD simulations.** **A.** RMSD plots of the sampled conformations from the initial energy-minimized structure for apo CRP monomer/dimer and holo CRP. **B.** The MSF of residues for all MD simulations. Subunits A, B of the dimer structures are plotted separately.
(TIF)

Figure S2 **Dynamic network in apo CRP monomer/dimer by parallel MD simulations.** The correlation between residue fluctuations based on first ten essential modes is presented for three parallel MD runs of apo CRP monomer (**A**) and dimer (**B**).
(TIF)

Figure S3 **Time evolution of clusters for all MD simulations.** The cluster evolution in time for the MD simulations of apo CRP monomer (three runs) (**A–C**), apo CRP dimer (three runs) (**D–F**), and holo CRP dimer (**G**) on the RMSD plot with a cluster radius of 3.5 Å.
(TIF)

Figure S4 **GNM cross-correlations of MD sampled conformational states.** The GNM correlations between residue fluctuations in the average ten slowest modes for the cluster best members of three apo monomer (**A, B, C**), three apo dimer (**D, E, F**) and a holo dimer (**G**) MD runs (with a cluster radius 3.5 Å). On the right, the ribbon diagrams color coded with the correlation values of the L134-D138 hinge (average) with the rest of the structure are given.
(DOCX)

Figure S5 **GNM cross-correlations of apo CRP NMR structures.** The GNM correlations between residue fluctuations in the average ten slowest modes for the twenty apo CRP NMR models (PDB: 2WC2). On the right, the ribbon diagrams color coded with the correlation values of the L134-D138 region (average) with the rest of the structure are given.
(DOCX)

Figure S6 **Conservation profiles.** The evolutionary conservation profile of CRP calculated via the ConSurf Web server (http://consurf.tau.ac.il) is shown. The protein is colored according to their conservation grades using the color-coding bar. The predicted hinge residues are shown with spheres. Most variable to most conserved residues are colored in turquoise (score 1) to maroon (score 9).
(TIF)

Figure S7 **GNM slow mode shapes for various CRP conformations.** The average three slowest GNM mode shapes for apo CRP NMR models (**A**), apo CRP monomer cluster best members (**B**), apo CRP dimer cluster best members (**C**), holo CRP crystal structures (**D**), and holo CRP cluster best members (**E**). (**F**) displays the hinges/flexible segments predicted by the GNM slow modes on an apo CRP monomer snapshot.
(TIF)
