# Supplementary figures and images for: Dynamic Fluctuations Provide the Basis of a Conformational Switch Mechanism in Apo Cyclic AMP Receptor Protein
Source: PLoS Comput Biol. 2013 Jul 18;9(7):e1003141. doi: 10.1371/journal.pcbi.1003141 (PMC3715548; doi:10.1371/journal.pcbi.1003141)

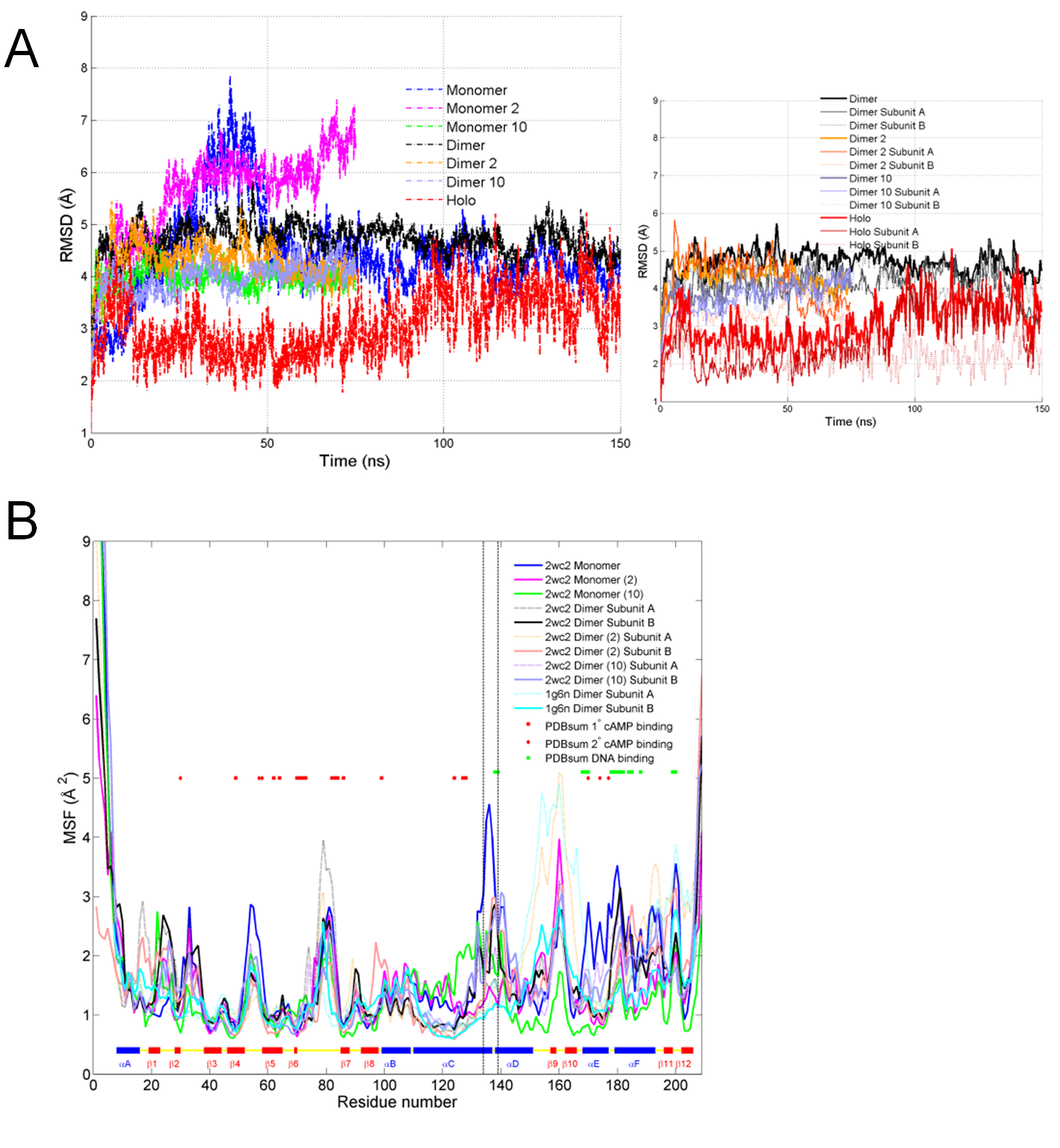

Supplement: Figure S1 — Fluctuations of apo CRP monomer/dimer and holo CRP by MD simulations. A. RMSD plots of the sampled conformations from the initial energy-minimized structure for apo CRP monomer/dimer and holo CRP. B. The MSF of residues for all MD simulations. Subunits A, B of the dimer structures are plotted separately. (TIF) [file pcbi.1003141.s001.tif]

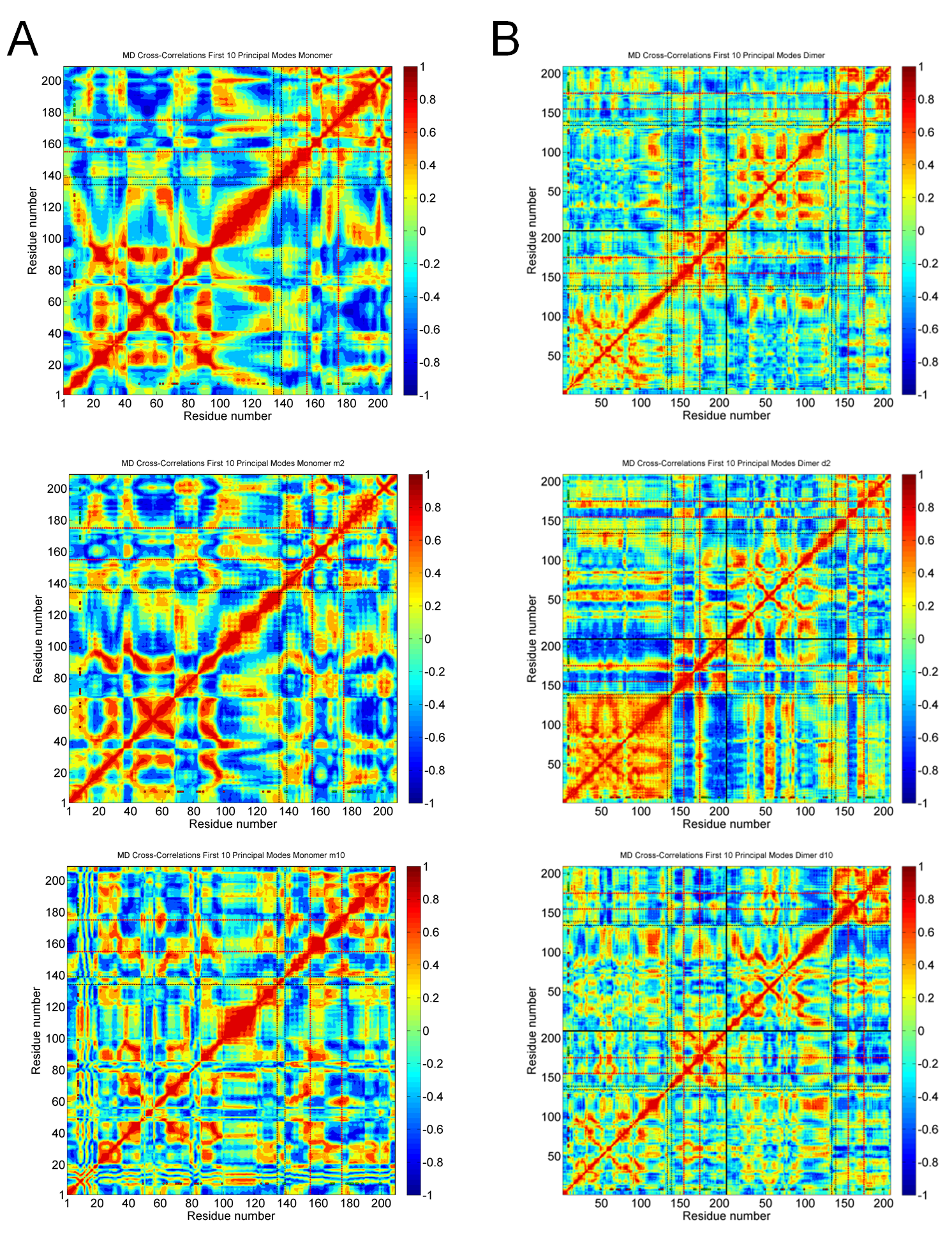

Supplement: Figure S2 — Dynamic network in apo CRP monomer/dimer by parallel MD simulations. The correlation between residue fluctuations based on first ten essential modes is presented for three parallel MD runs of apo CRP monomer (A) and dimer (B). (TIF) [file pcbi.1003141.s002.tif]

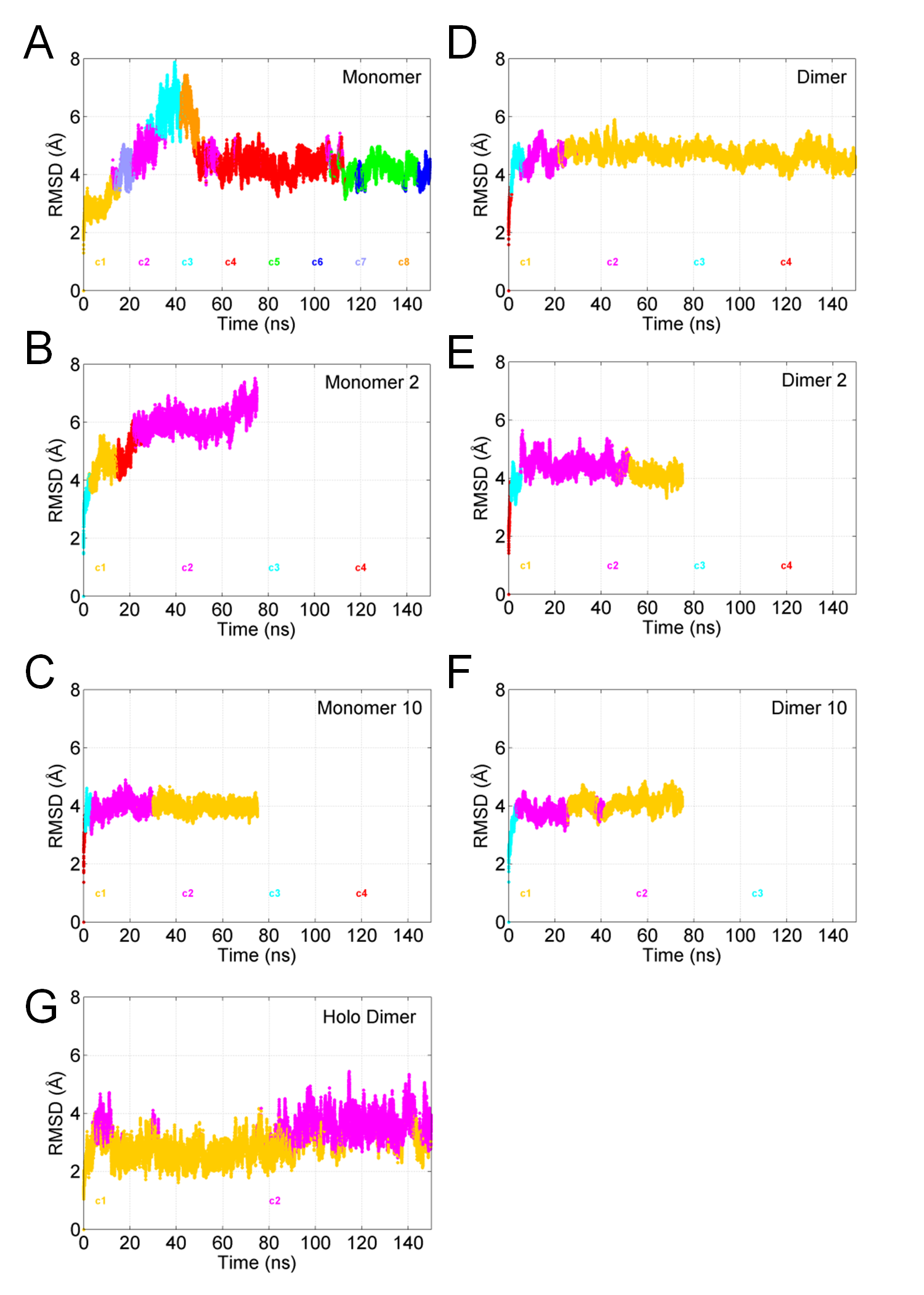

Supplement: Figure S3 — Time evolution of clusters for all MD simulations. The cluster evolution in time for the MD simulations of apo CRP monomer (three runs) (A–C), apo CRP dimer (three runs) (D–F), and holo CRP dimer (G) on the RMSD plot with a cluster radius of 3.5 Å. (TIF) [file pcbi.1003141.s003.tif]

| 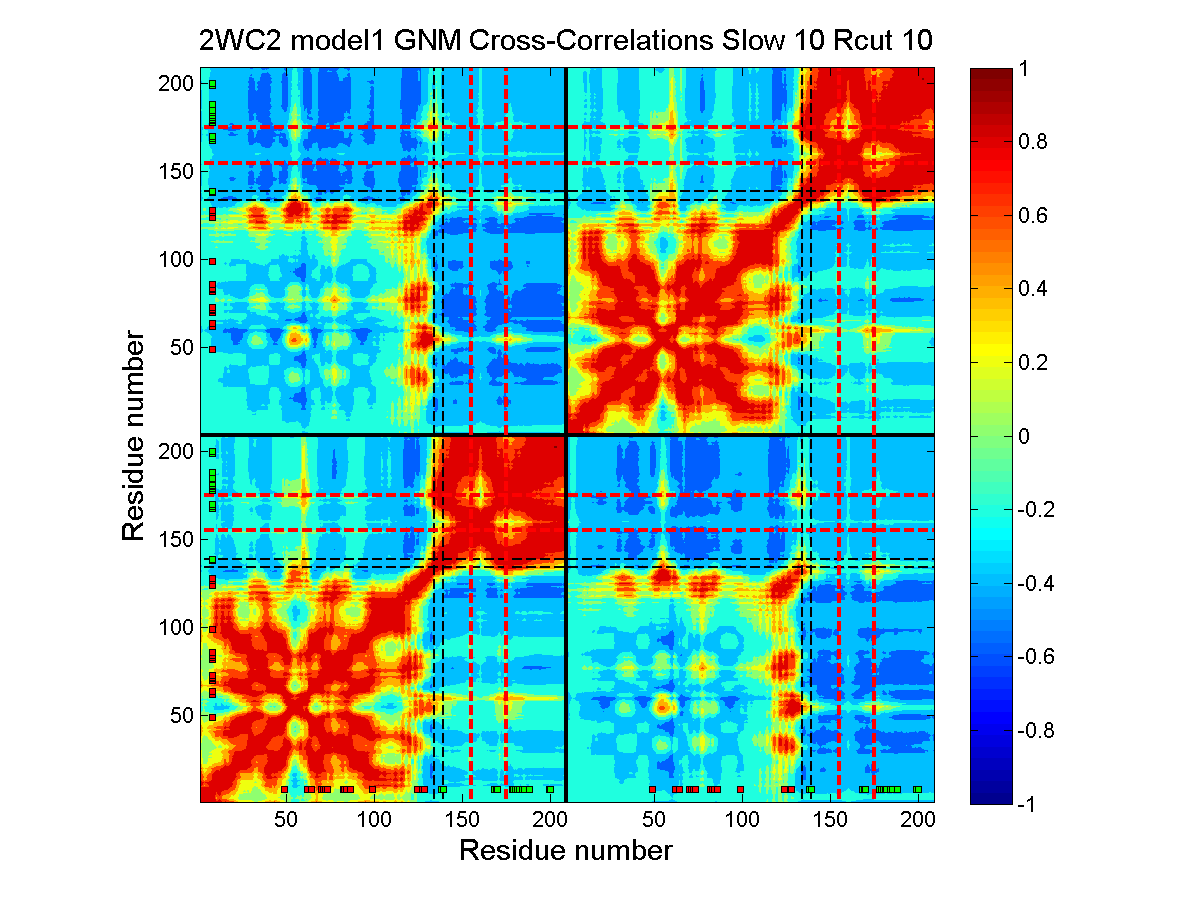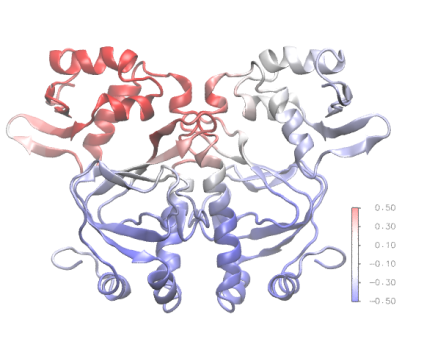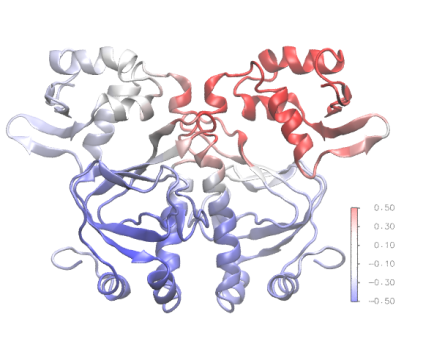 | 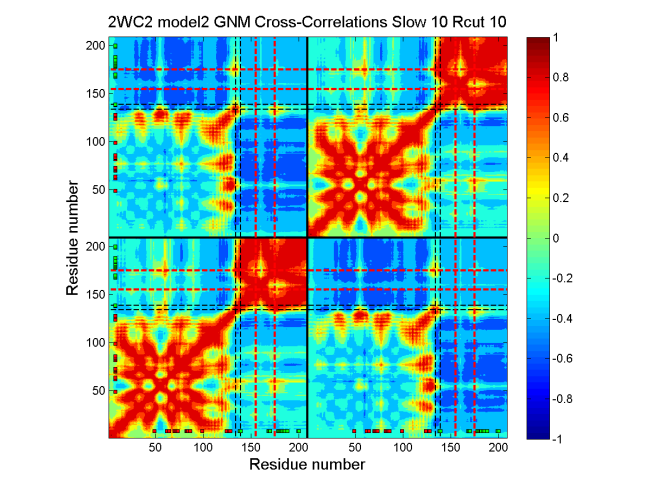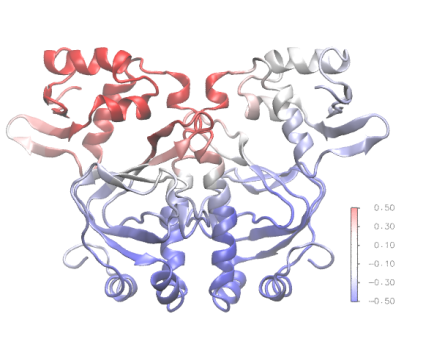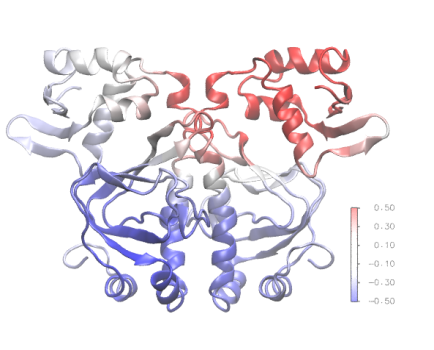 | 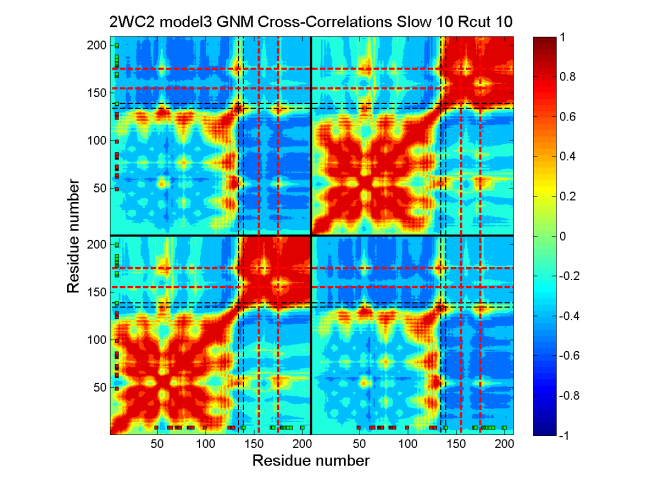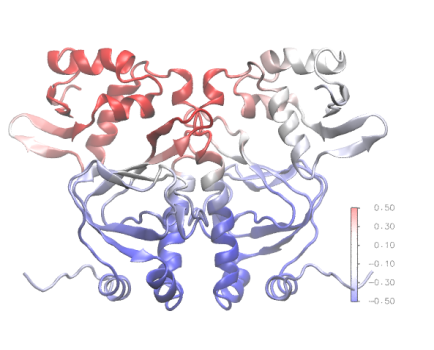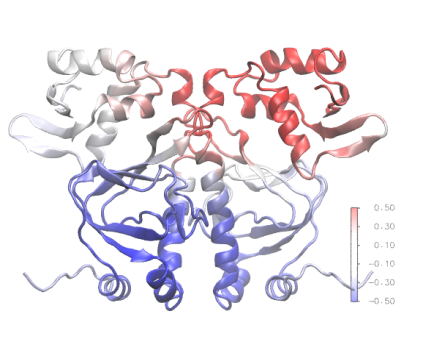 | 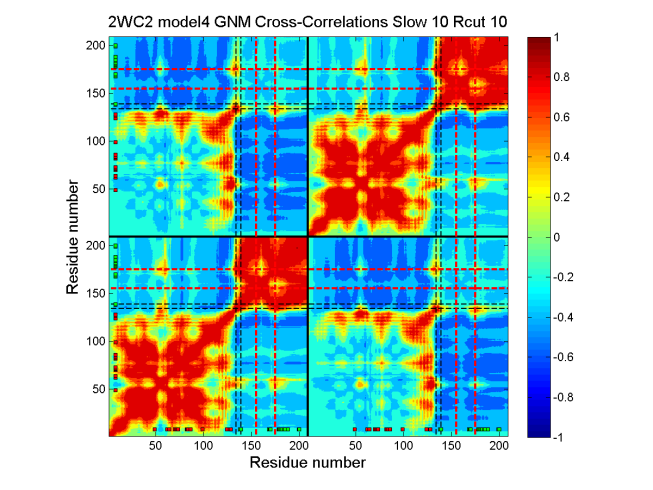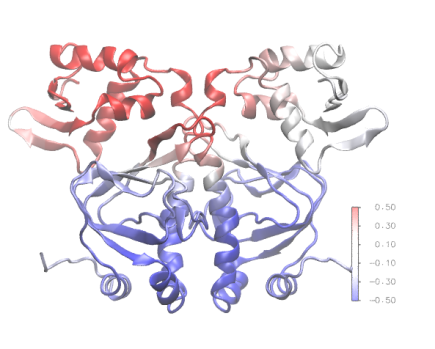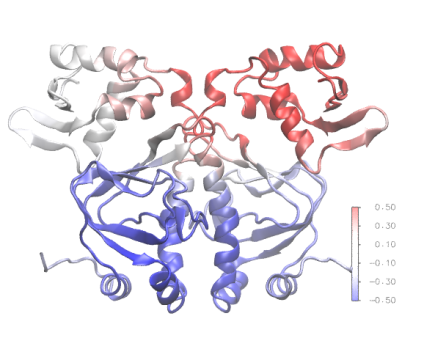 |
| --- | --- | --- | --- |
| 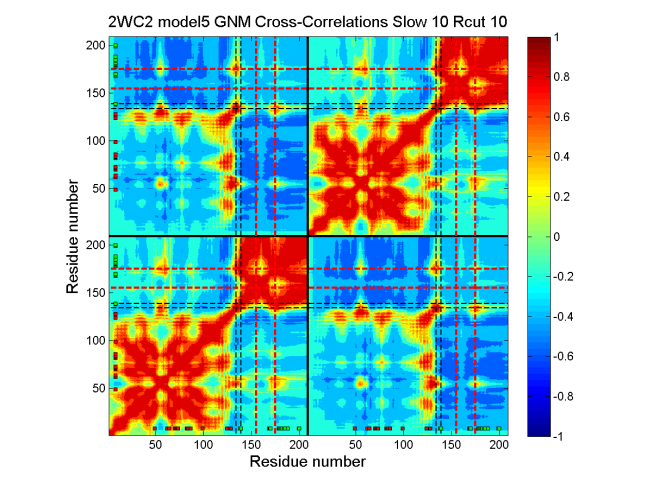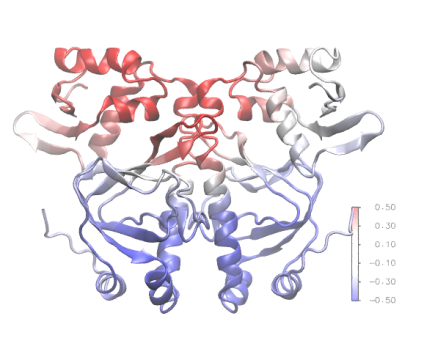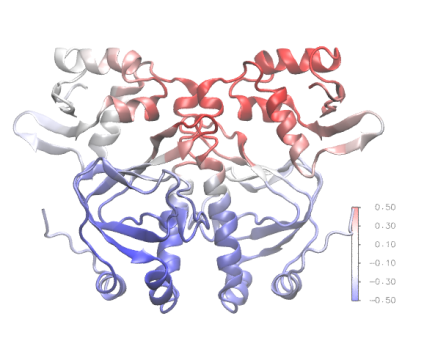 | 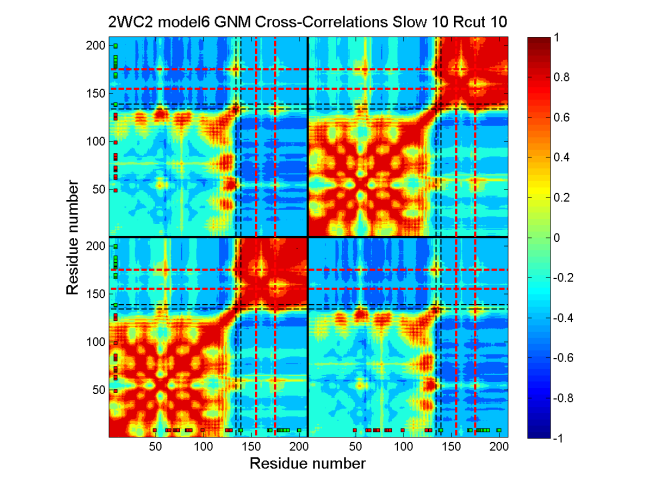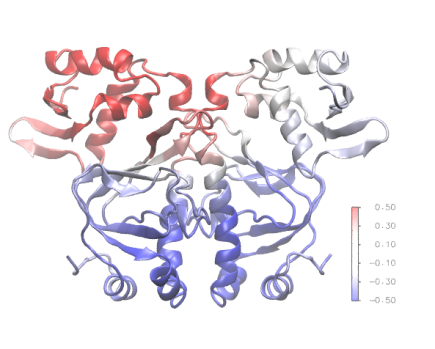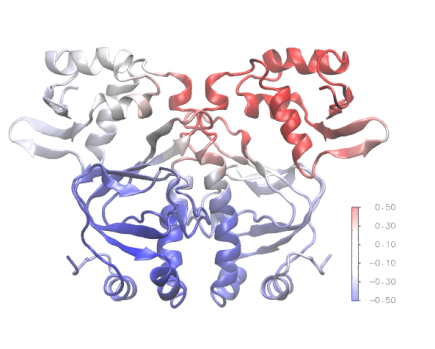 | 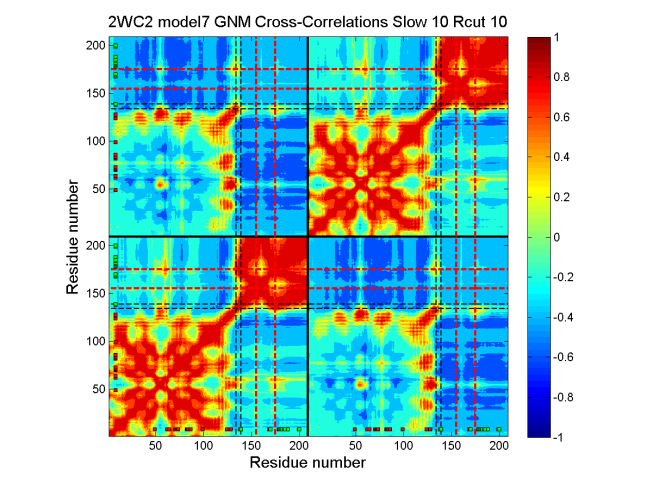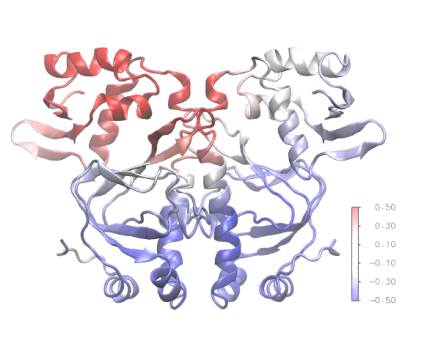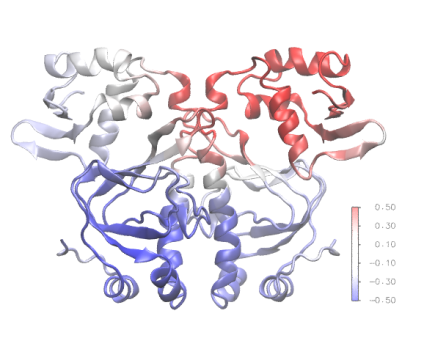 | 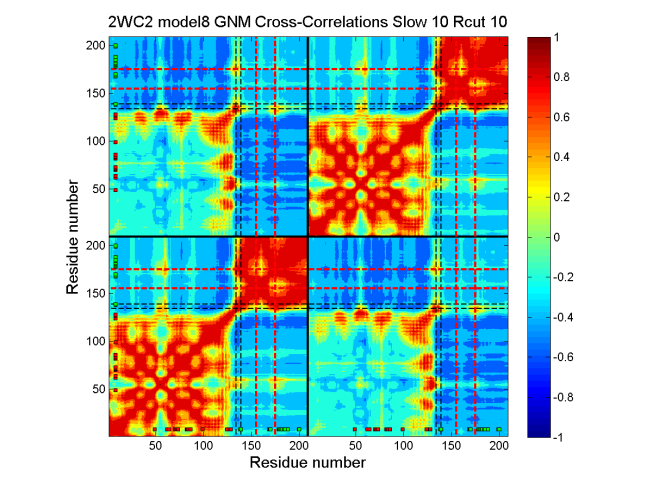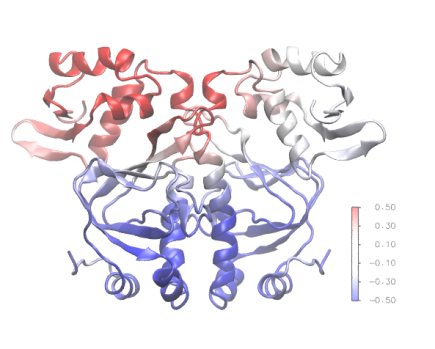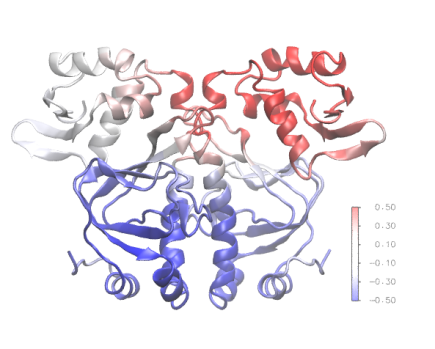 |
|  |  |  |  |
| 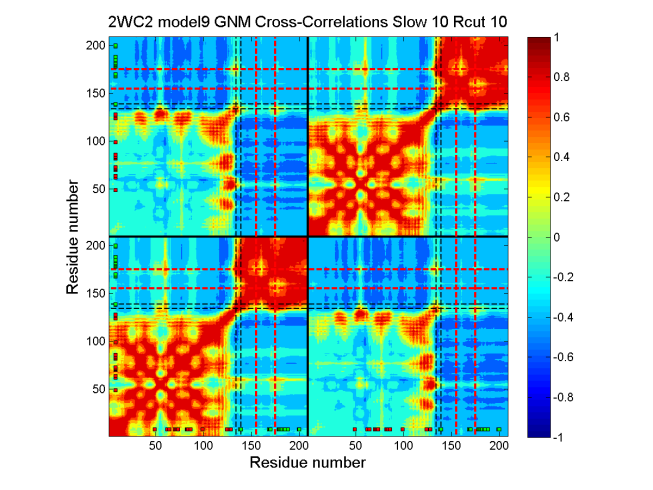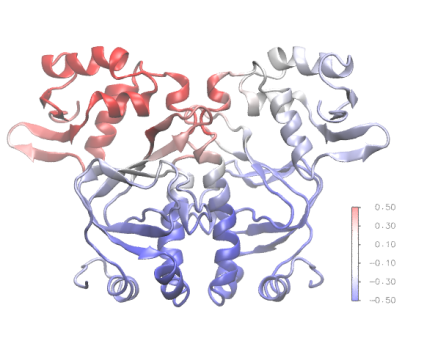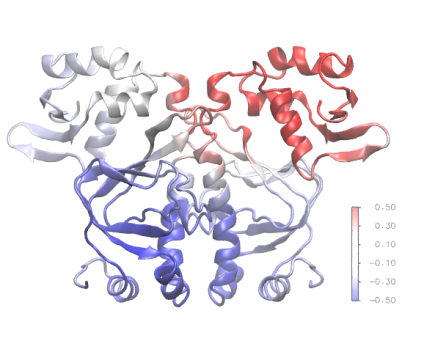 | 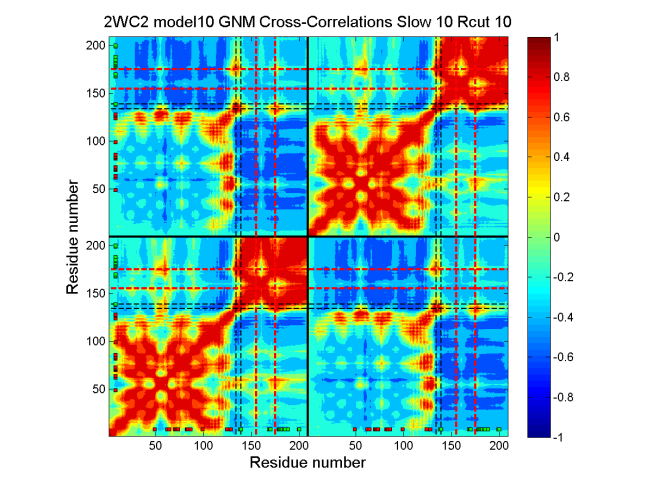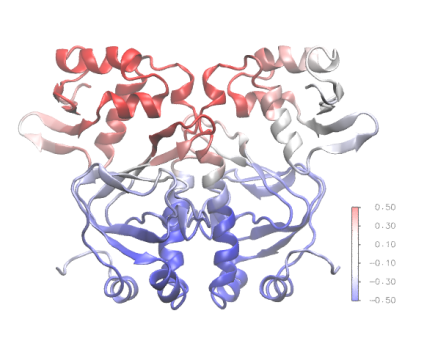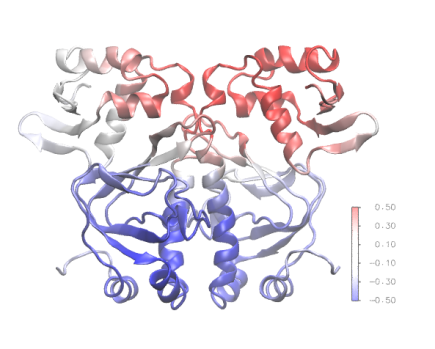 | 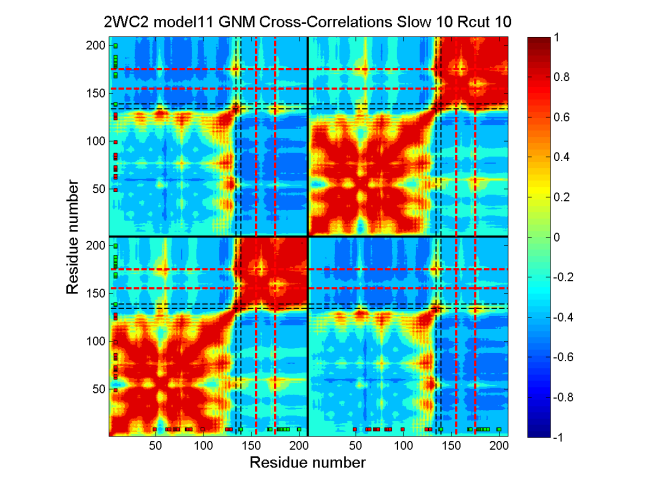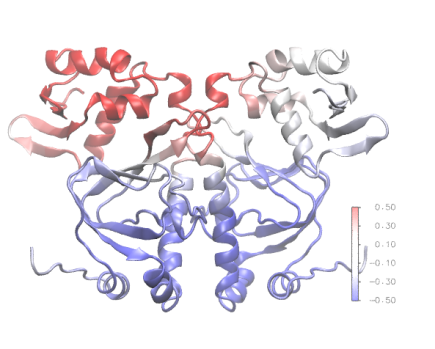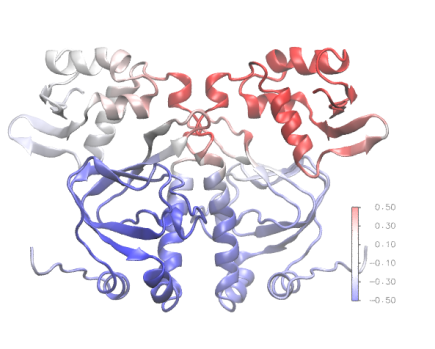 | 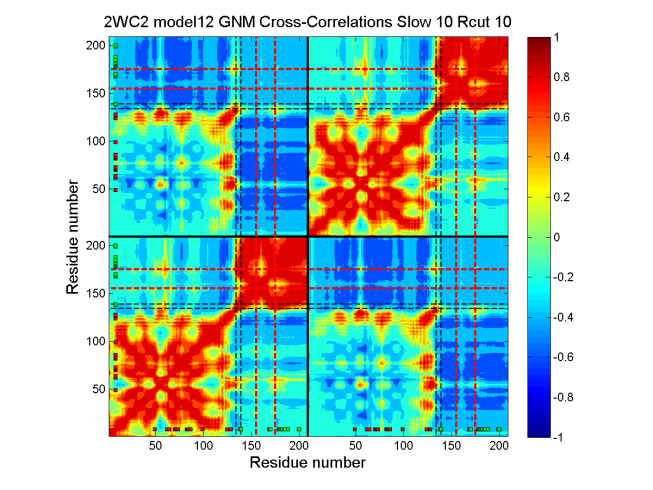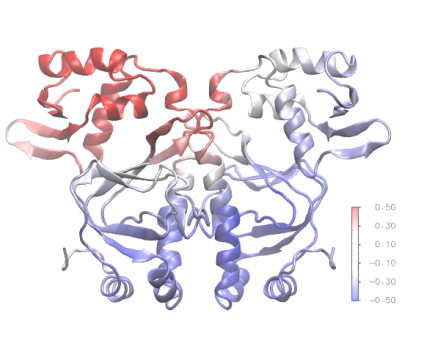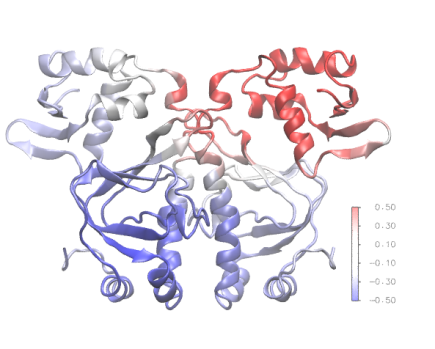 |
| 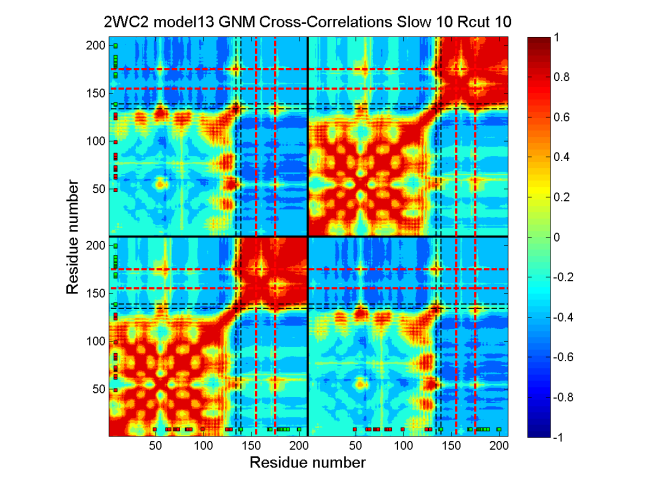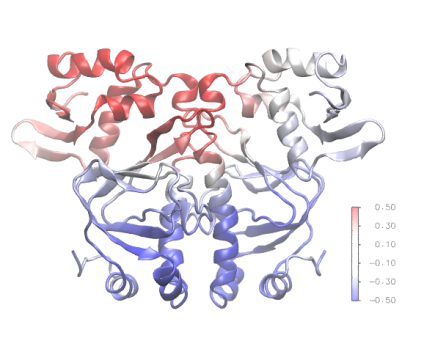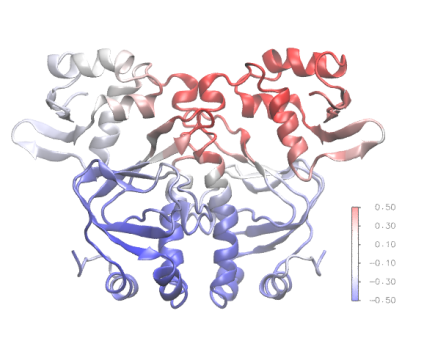 | 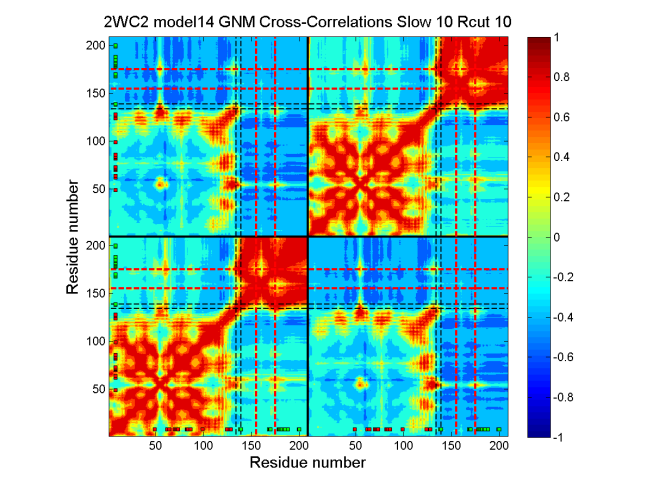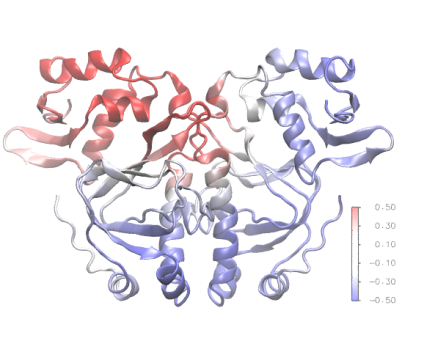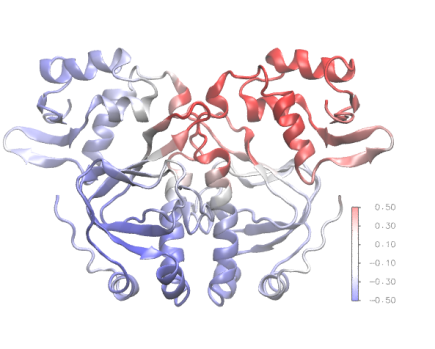 | 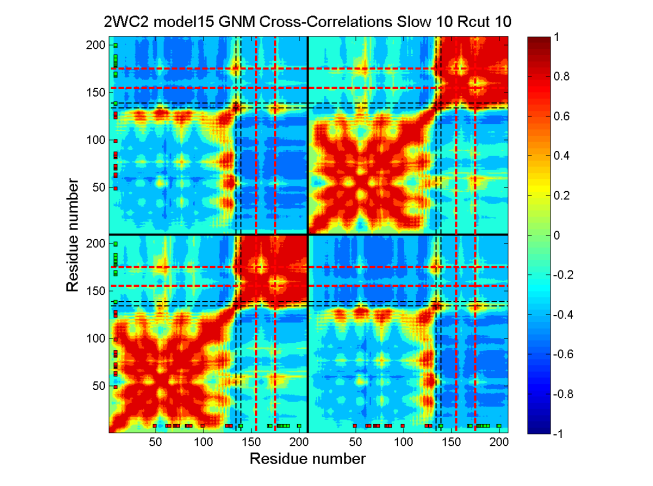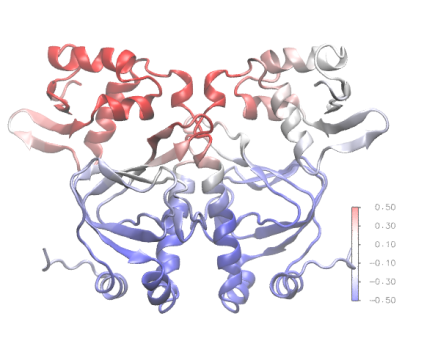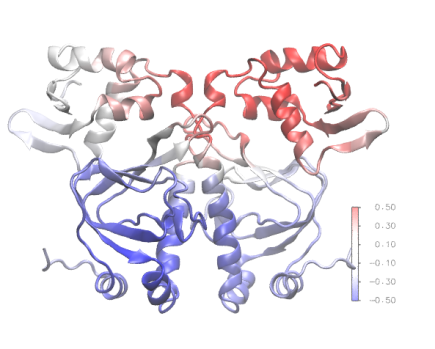 | 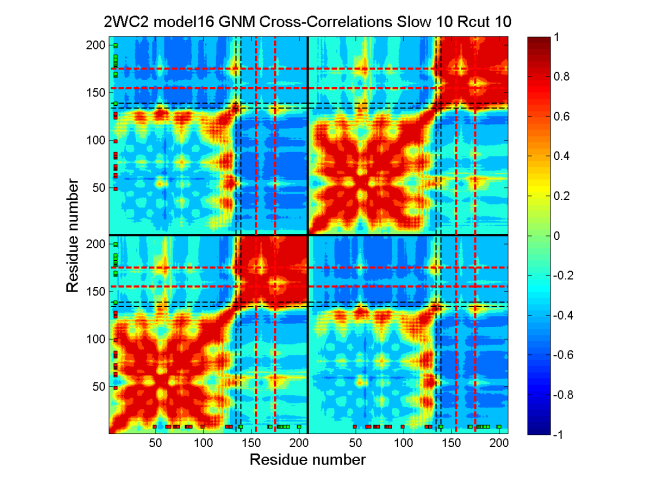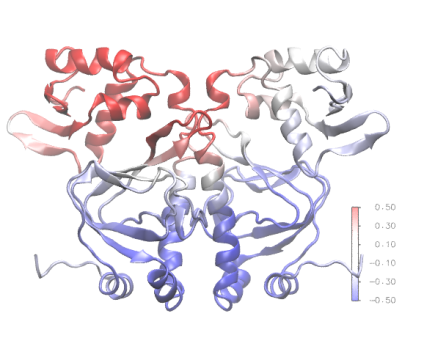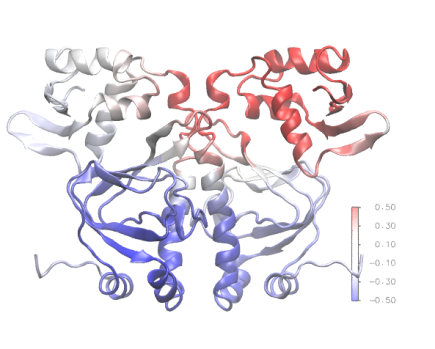 |
| 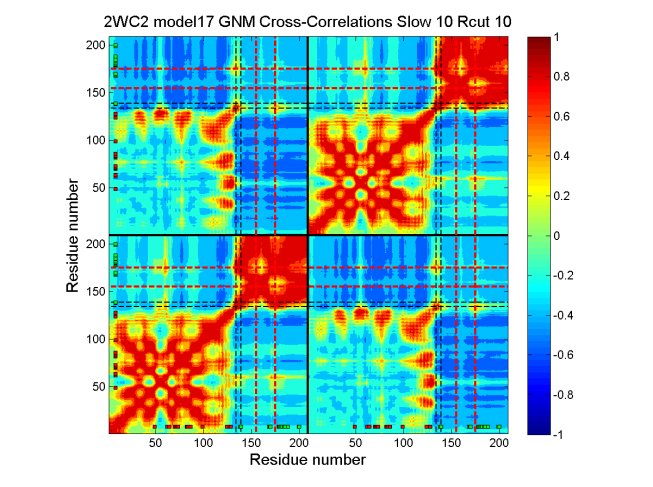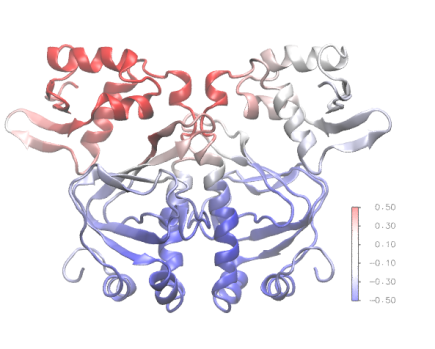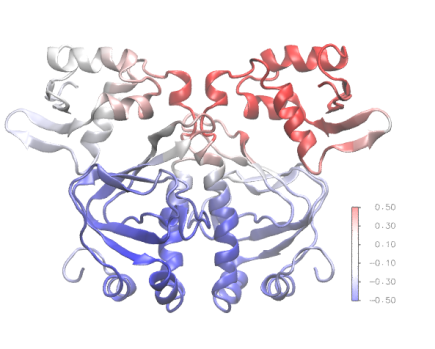 | 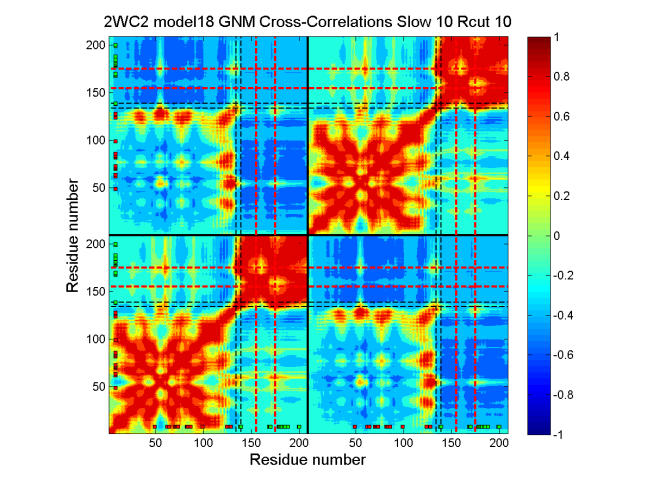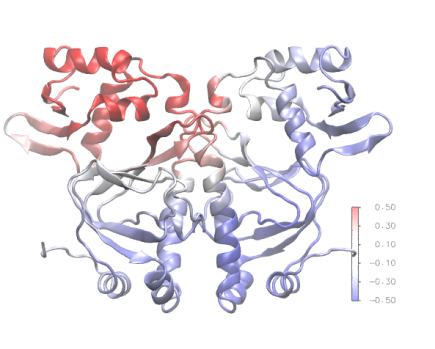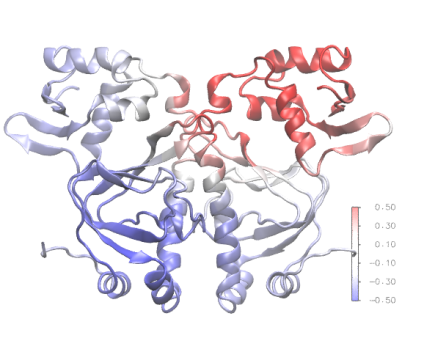 | 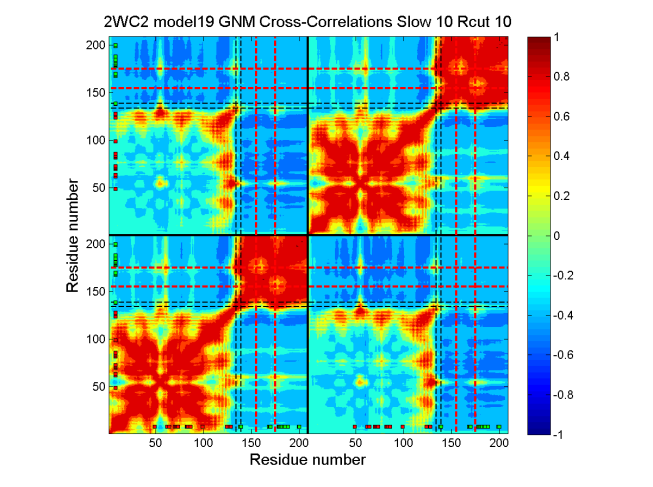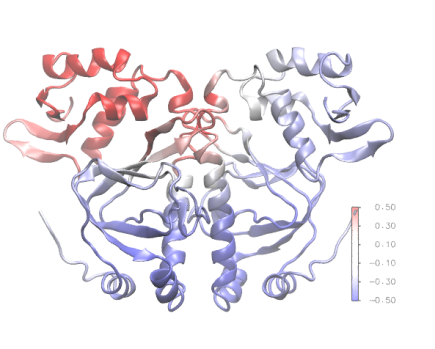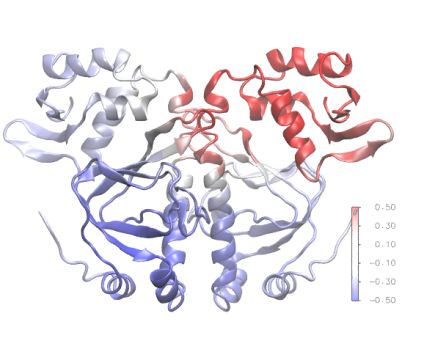 | 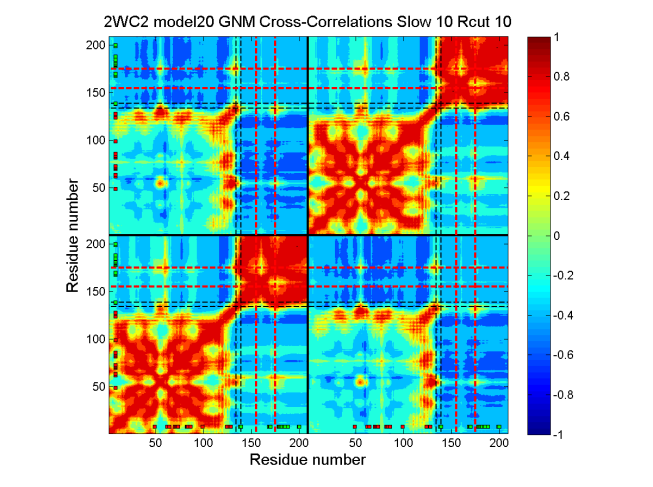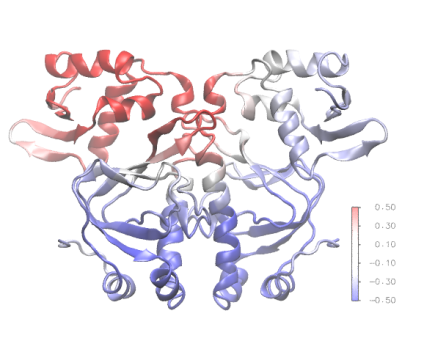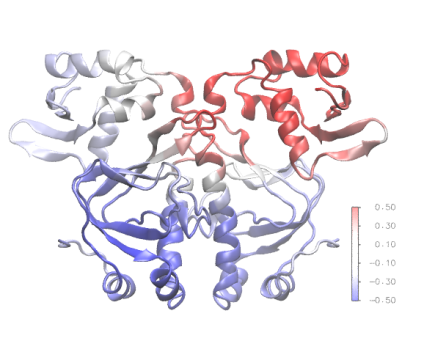 |

Supplement: Figure S5 — GNM cross-correlations of apo CRP NMR structures. The GNM correlations between residue fluctuations in the average ten slowest modes for the twenty apo CRP NMR models (PDB: 2WC2). On the right, the ribbon diagrams color coded with the correlation values of the L134-D138 region (average) with the rest of the structure are given. (DOCX) [file pcbi.1003141.s005.docx]

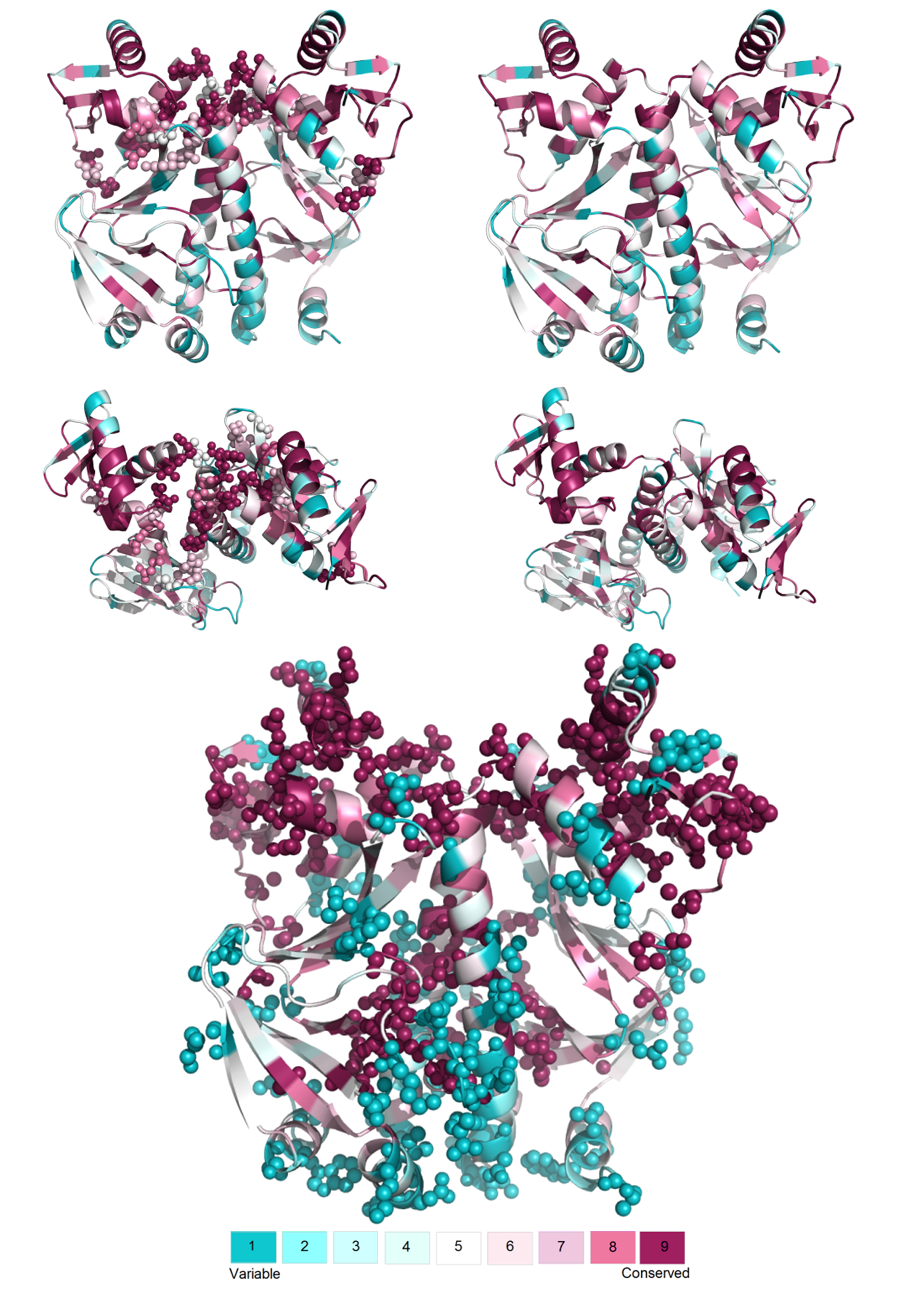

Supplement: Figure S6 — Conservation profiles. The evolutionary conservation profile of CRP calculated via the ConSurf Web server (http://consurf.tau.ac.il) is shown. The protein is colored according to their conservation grades using the color-coding bar. The predicted hinge residues are shown with spheres. Most variable to most conserved residues are colored in turquoise (score 1) to maroon (score 9). (TIF) [file pcbi.1003141.s006.tif]

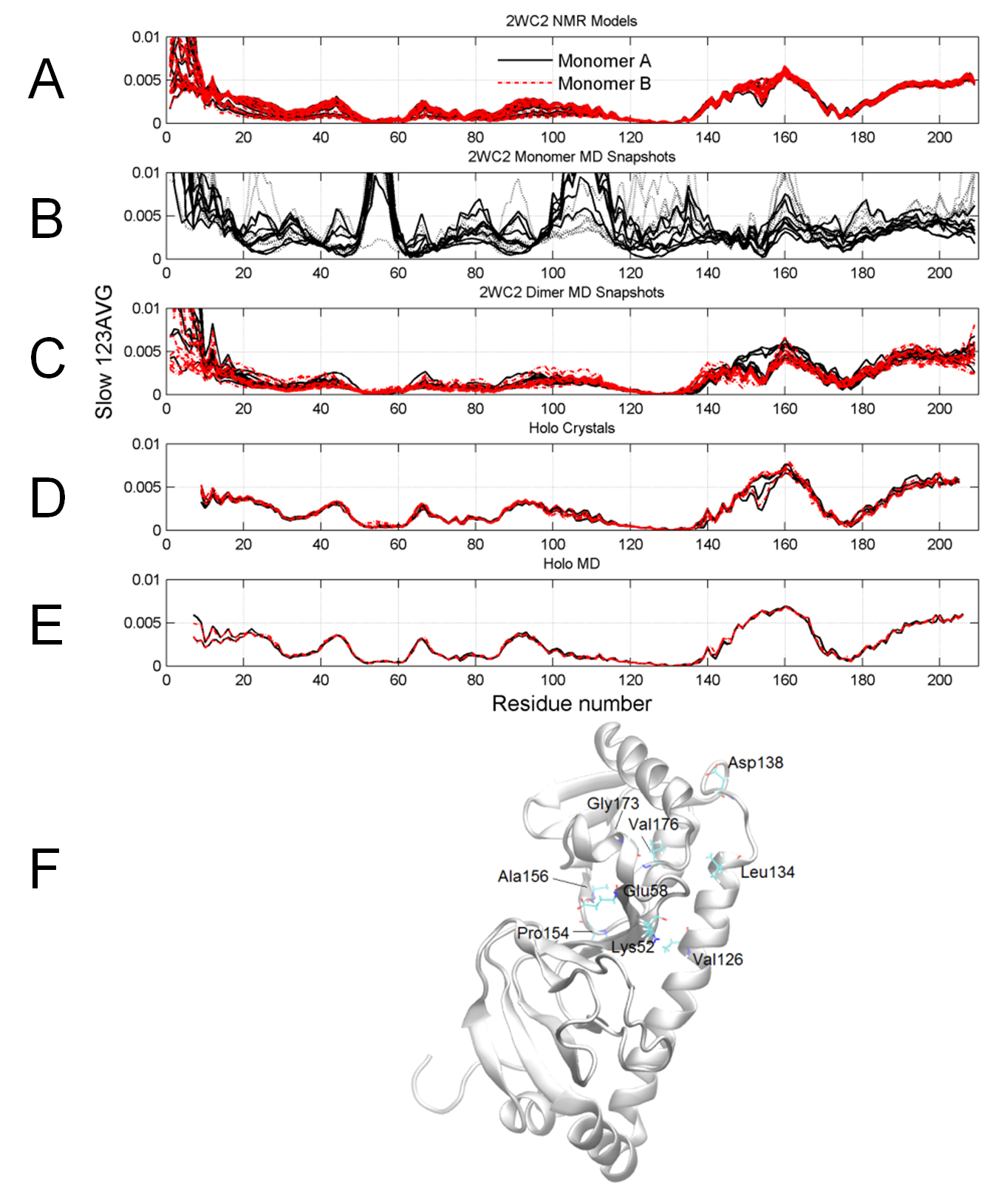

Supplement: Figure S7 — GNM slow mode shapes for various CRP conformations. The average three slowest GNM mode shapes for apo CRP NMR models (A), apo CRP monomer cluster best members (B), apo CRP dimer cluster best members (C), holo CRP crystal structures (D), and holo CRP cluster best members (E). (F) displays the hinges/flexible segments predicted by the GNM slow modes on an apo CRP monomer snapshot. (TIF) [file pcbi.1003141.s007.tif]
