# Supplementary material for: Dynamic Fluctuations Provide the Basis of a Conformational Switch Mechanism in Apo Cyclic AMP Receptor Protein
Source: PLoS Comput Biol. 2013 Jul 18;9(7):e1003141. doi: 10.1371/journal.pcbi.1003141 (PMC3715548; doi:10.1371/journal.pcbi.1003141)
Supplement: Figure S4 — GNM cross-correlations of MD sampled conformational states. The GNM correlations between residue fluctuations in the average ten slowest modes for the cluster best members of three apo monomer (A, B, C), three apo dimer (D, E, F) and a holo dimer (G) MD runs (with a cluster radius 3.5 Å). On the right, the ribbon diagrams color coded with the correlation values of the L134-D138 hinge (average) with the rest of the structure are given. (DOCX) [file pcbi.1003141.s004.docx]

| 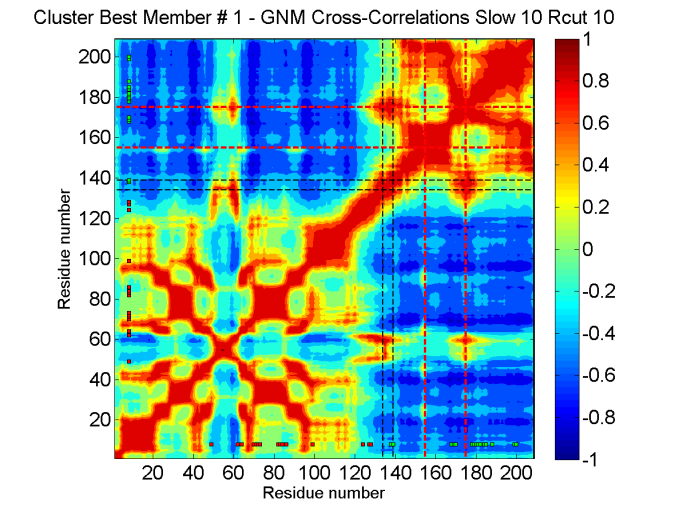  A | Cluster #1  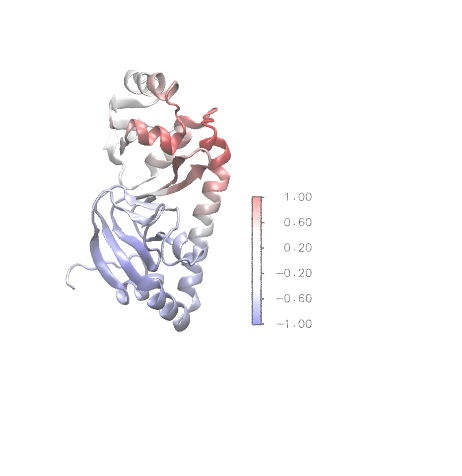  *t = 2 ns* | 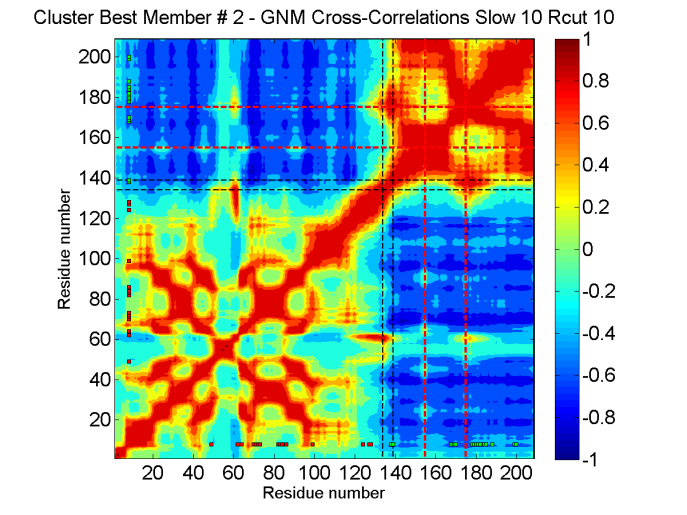 | Cluster #7  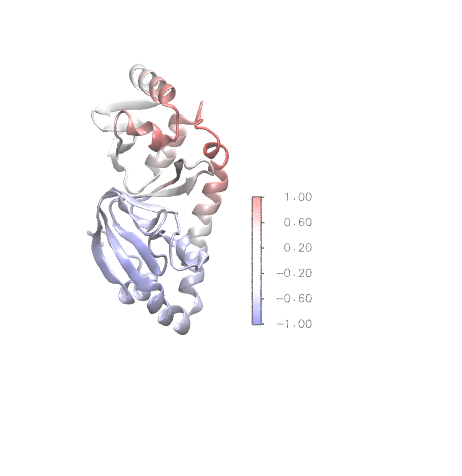  *t = 21 ns* |
| --- | --- | --- | --- |
| 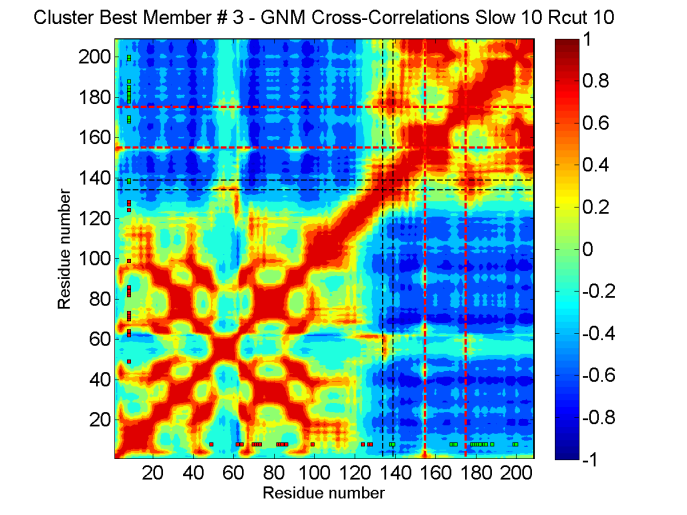 | Cluster #2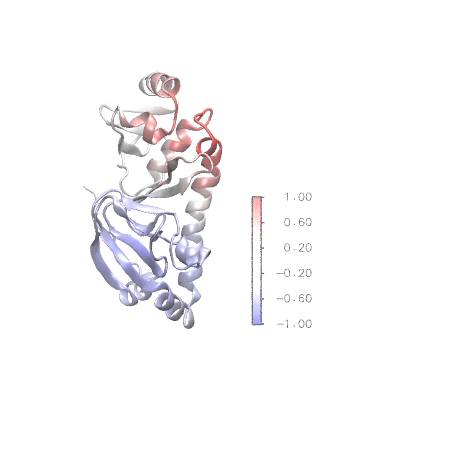  *t = 27 ns* | 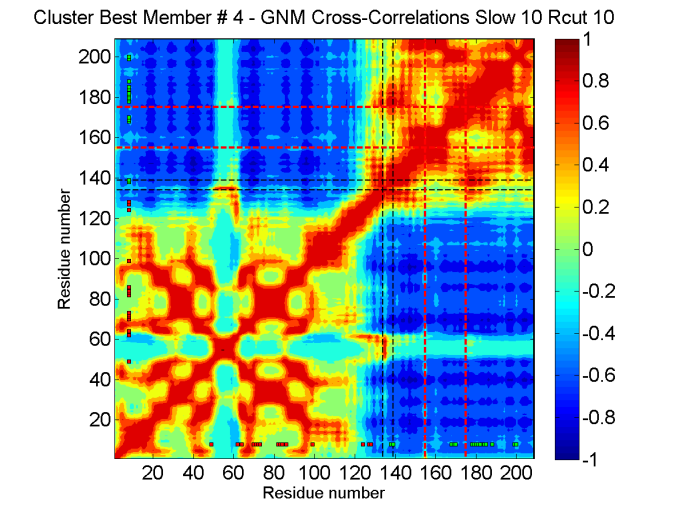 | Cluster #3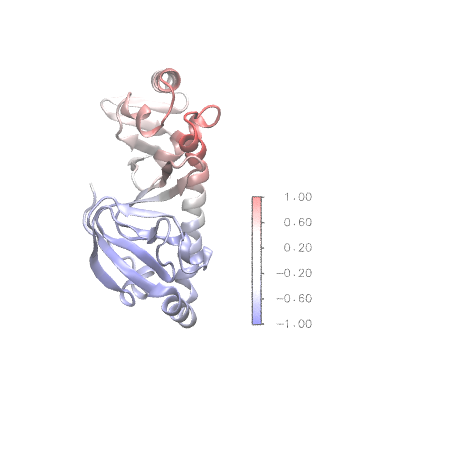  *t = 38 ns* |
| 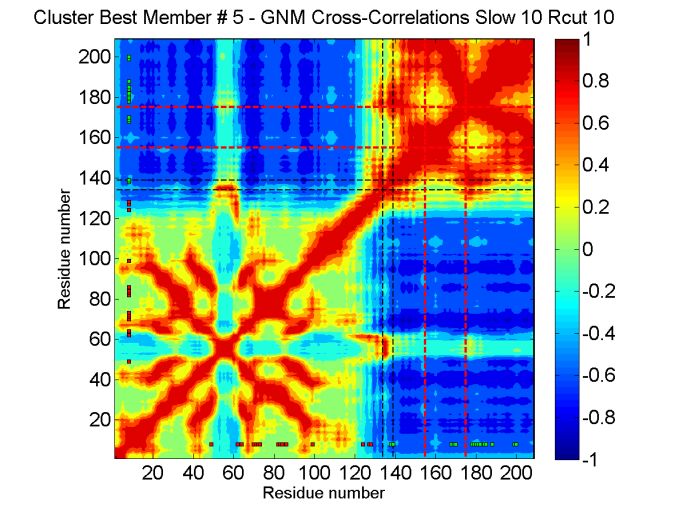 | Cluster #8  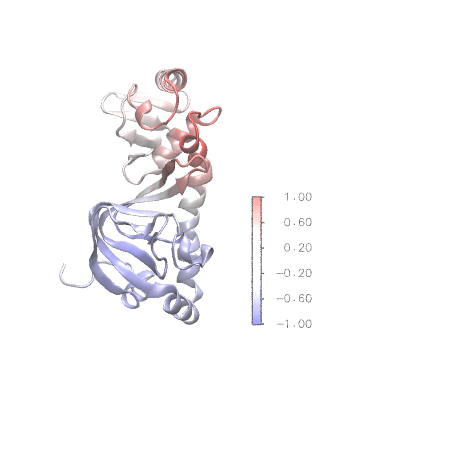  *t = 46 ns* | 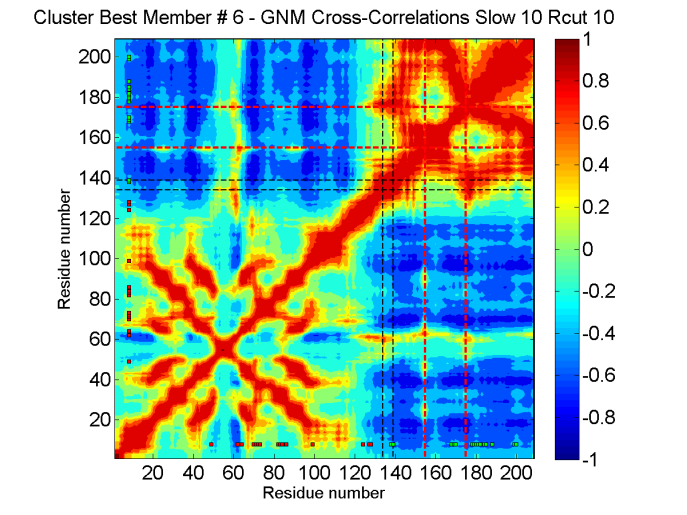 | Cluster #4  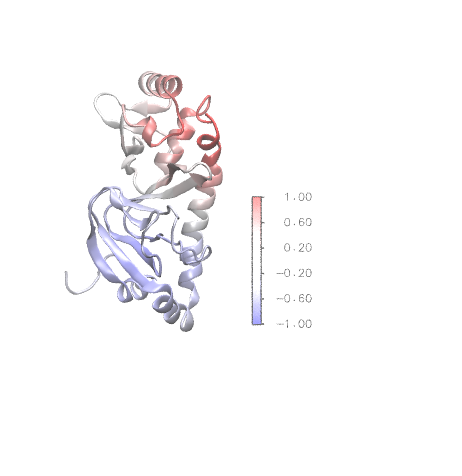  *t = 75 ns* |
| 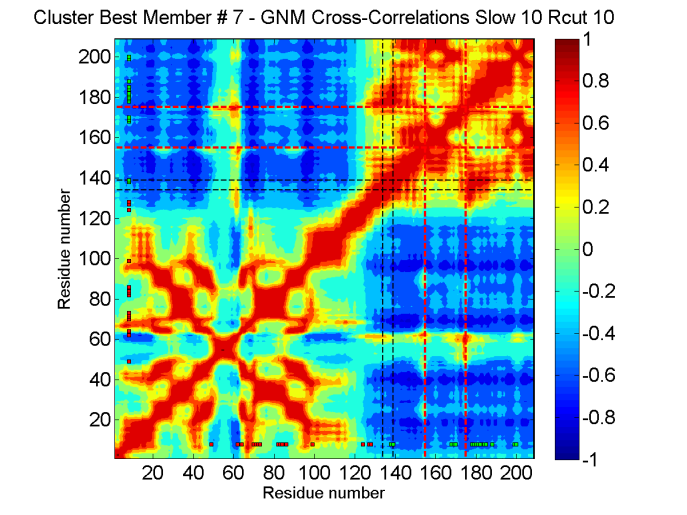 | Cluster #5  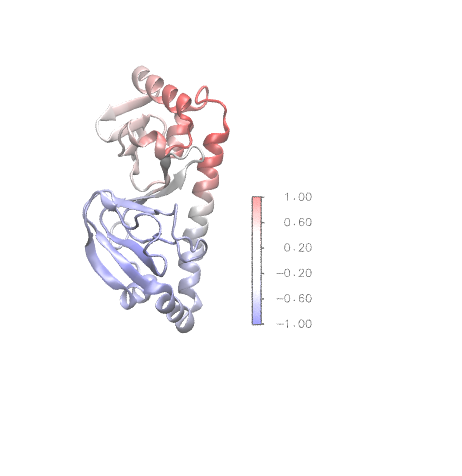  *t = 131 ns* | 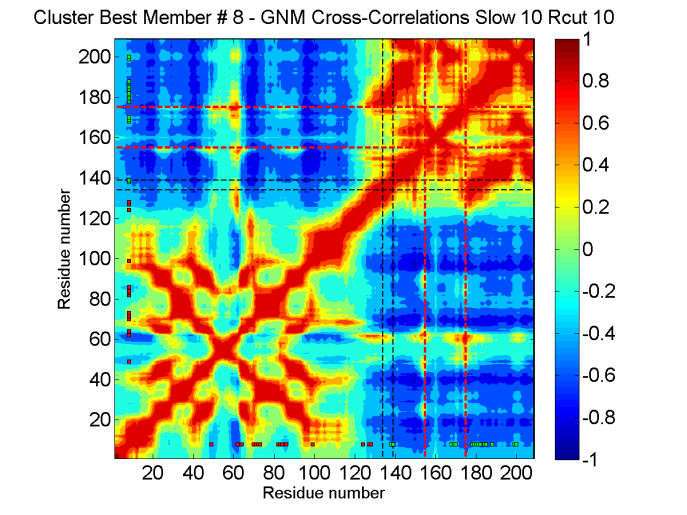 | Cluster #6  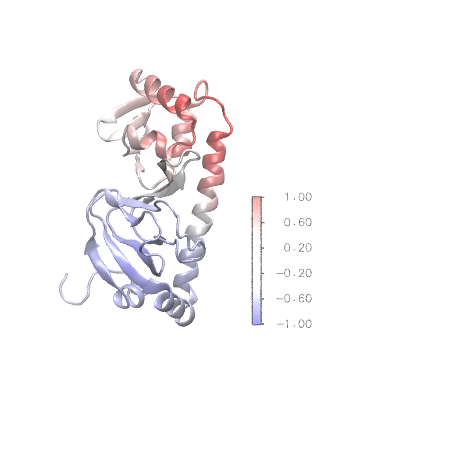  *t = 146 ns* |

| B  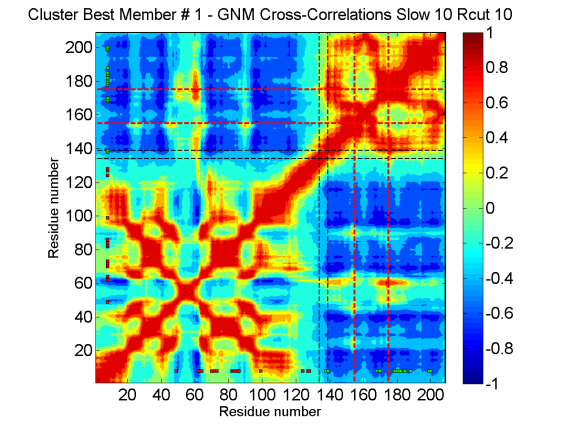 | Cluster #3  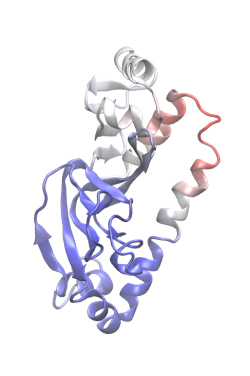  *t = 1.4 ns* | 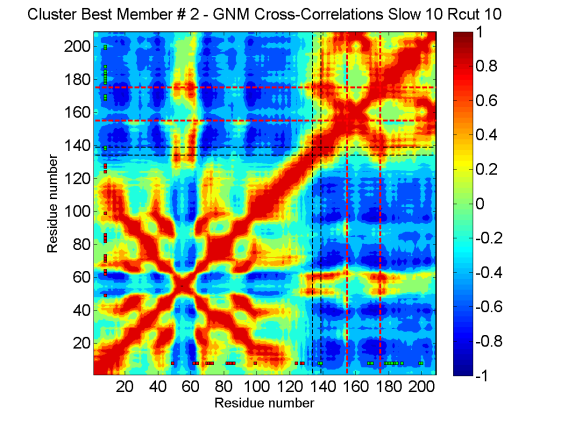 | Cluster #1  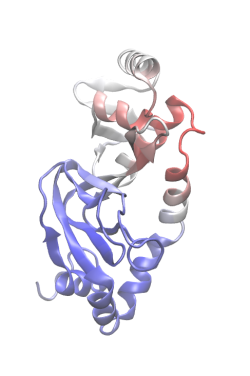  *t = 11.5 ns* |
| --- | --- | --- | --- |
| 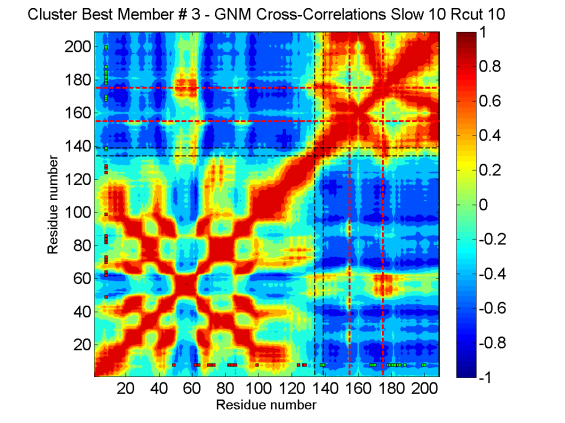 | Cluster #4  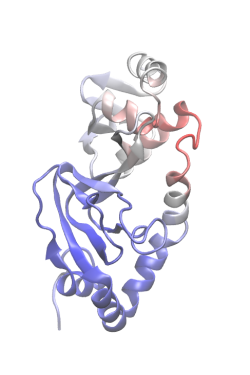  *t = 17.6 ns* | 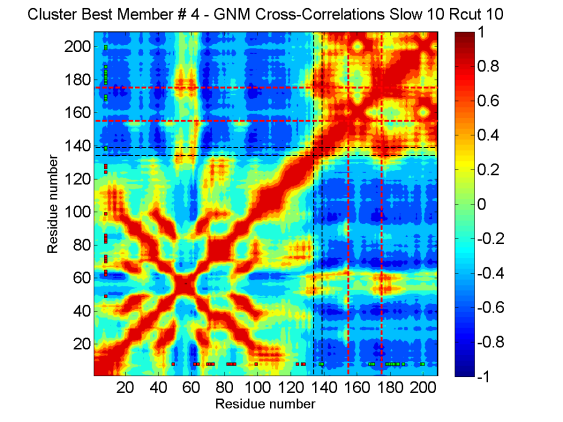 | Cluster #2  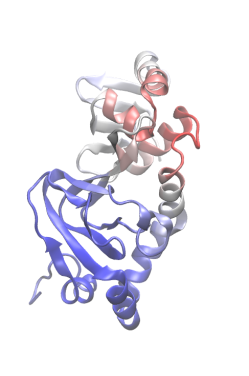  *t = 35.7 ns* |

C

| 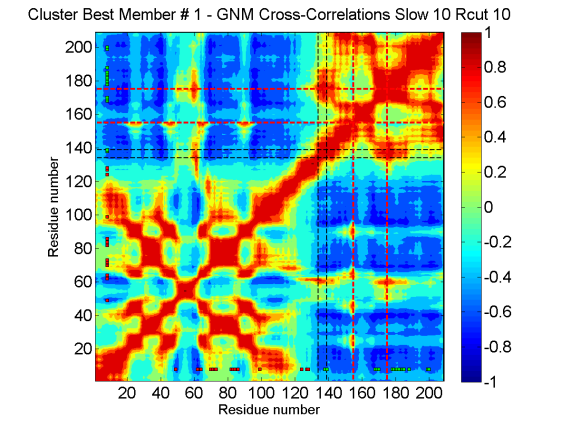 | Cluster #4  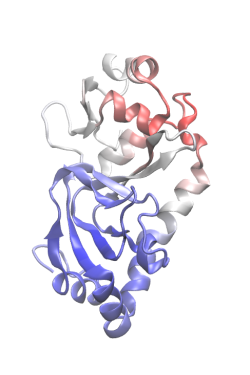  *t = 0.3 ns* | 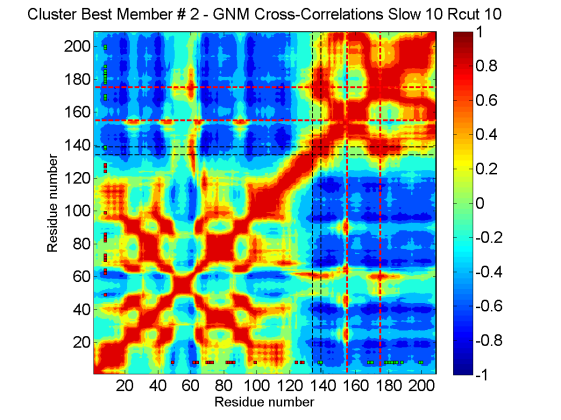 | Cluster #3  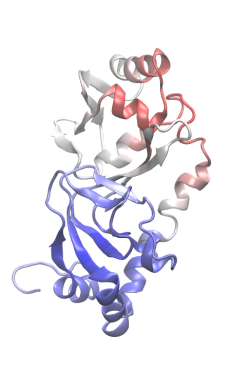  *t = 2.0 ns* |
| --- | --- | --- | --- |
| 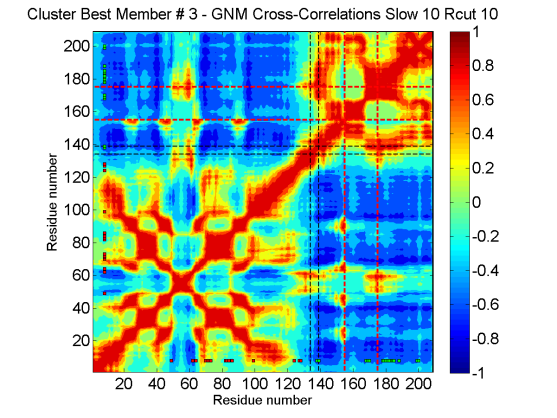 | Cluster #2  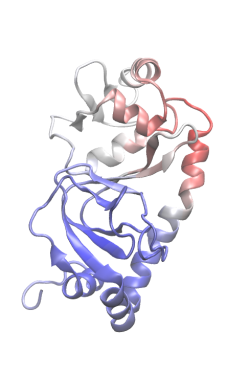  *t = 18.8 ns* | 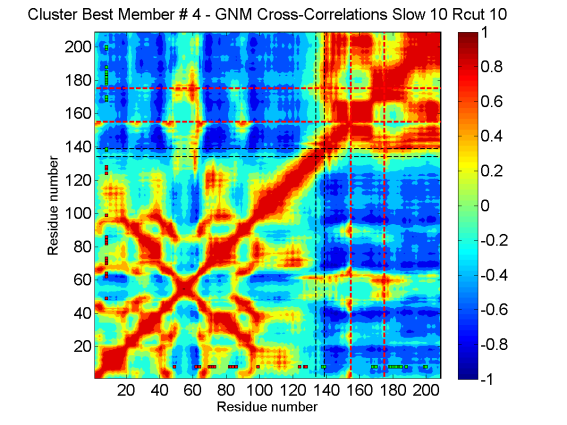 | Cluster #1  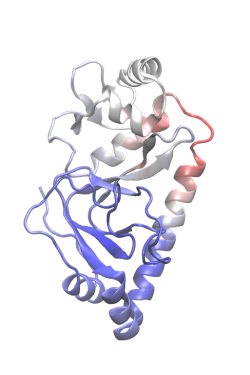  *t = 62.3 ns* |

| D  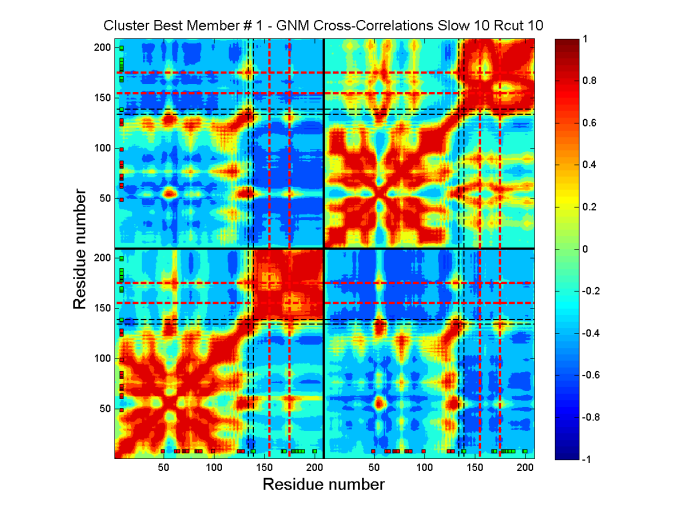  *t = 1 ns* | Cluster #4  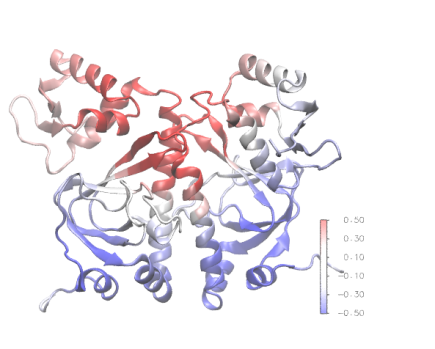  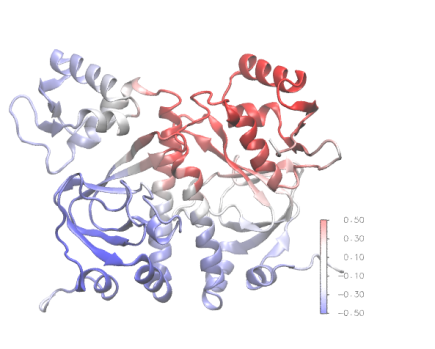 | 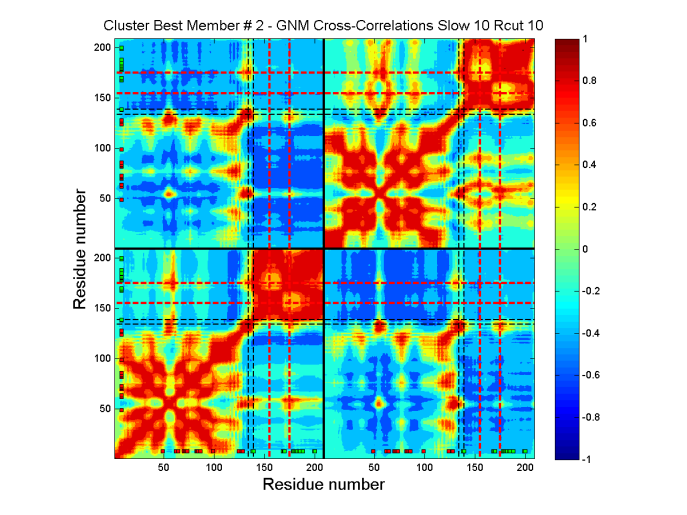  *t = 4 ns* | Cluster #3  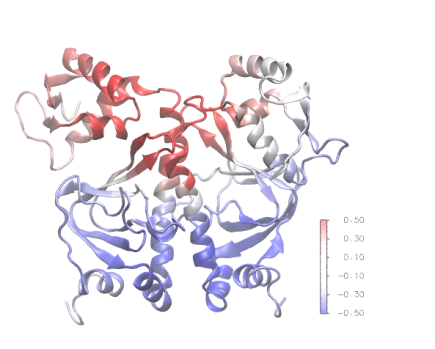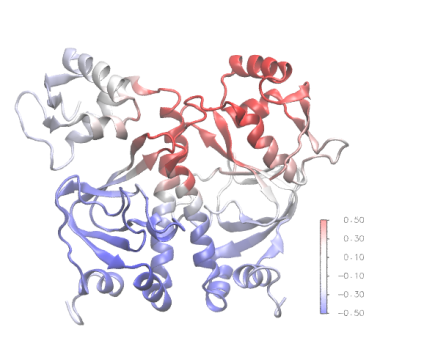 |
| --- | --- | --- | --- |
| 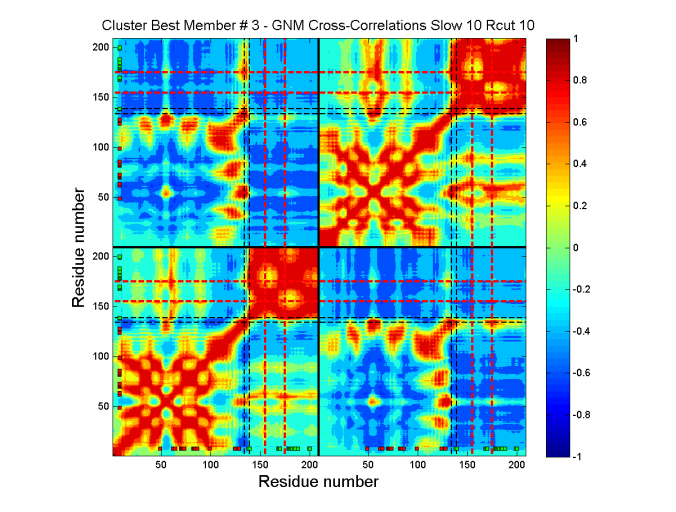  *t = 17 ns* | Cluster #2  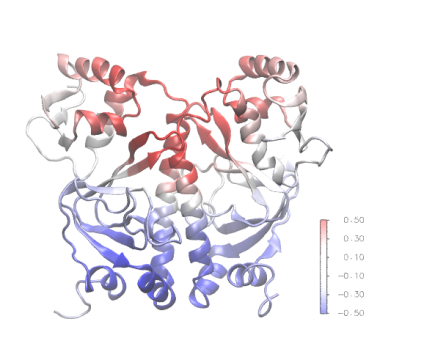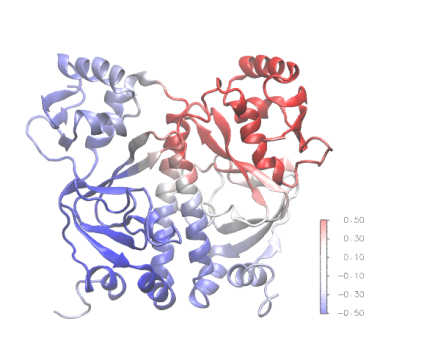 | 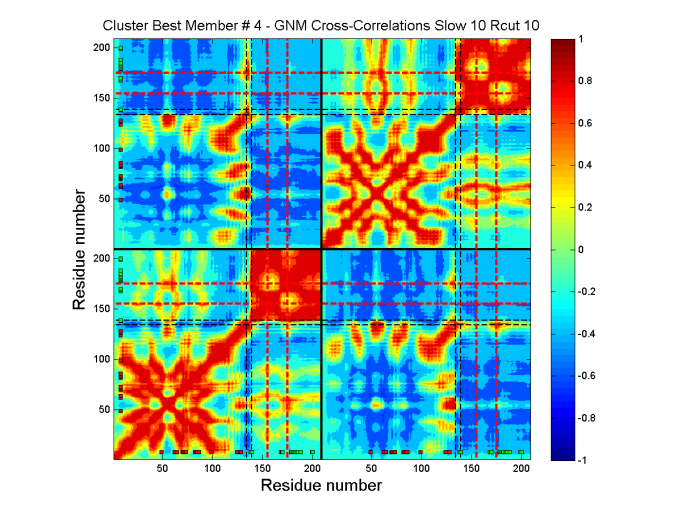  *t = 71 ns* | Cluster #1  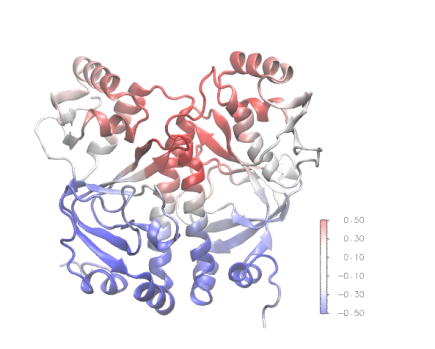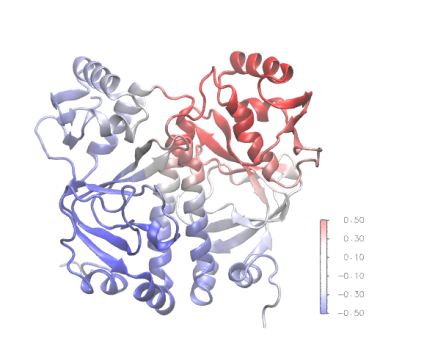 |

| E  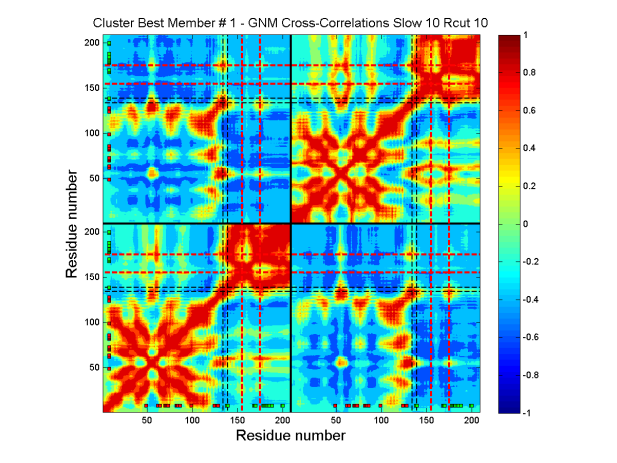  *t = 1 ns* | Cluster #4  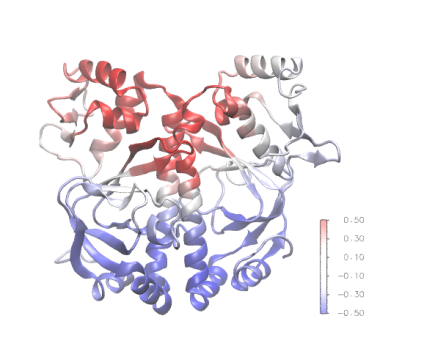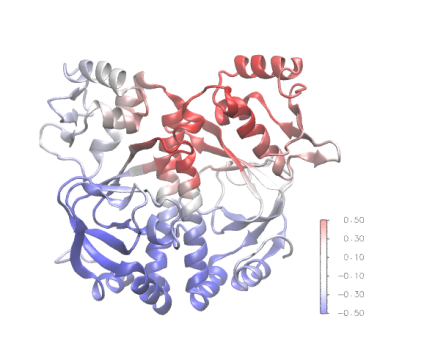 | 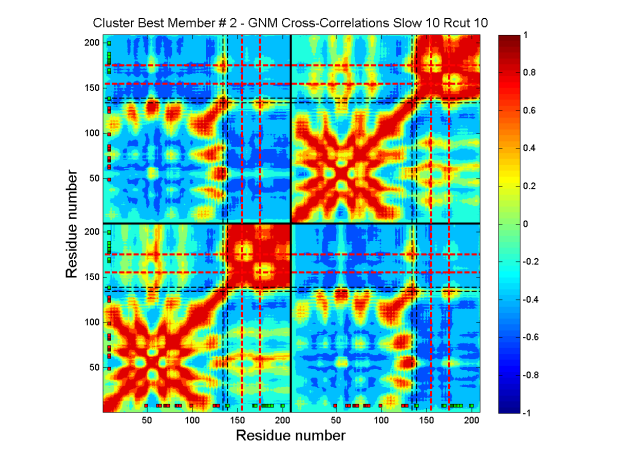  *t = 4 ns* | Cluster #3  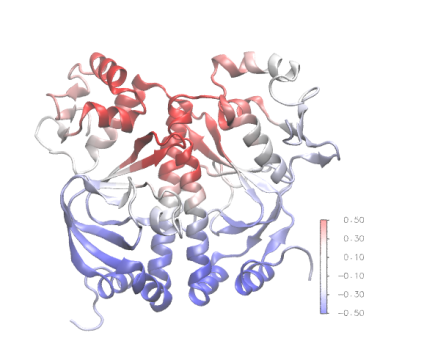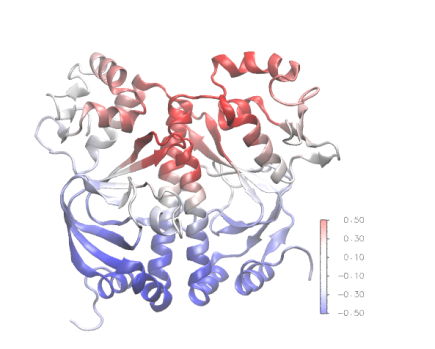 |
| --- | --- | --- | --- |
| 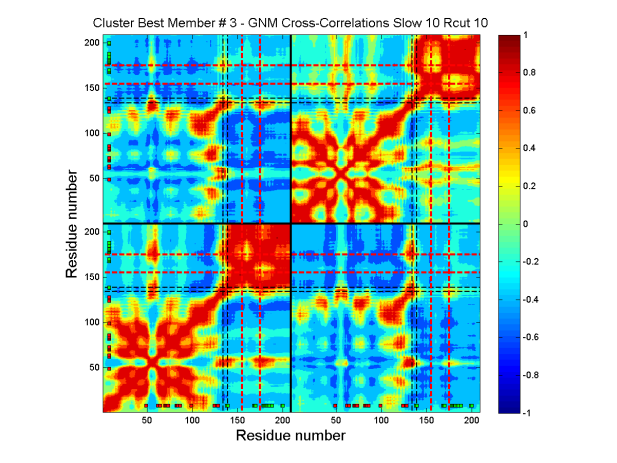  *t = 33 ns* | Cluster #2  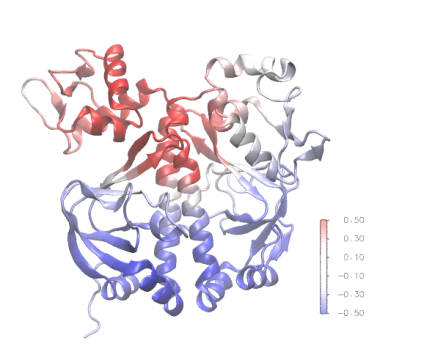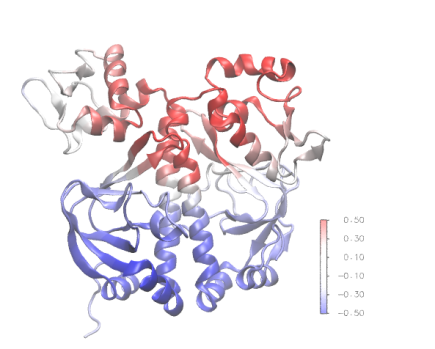 | 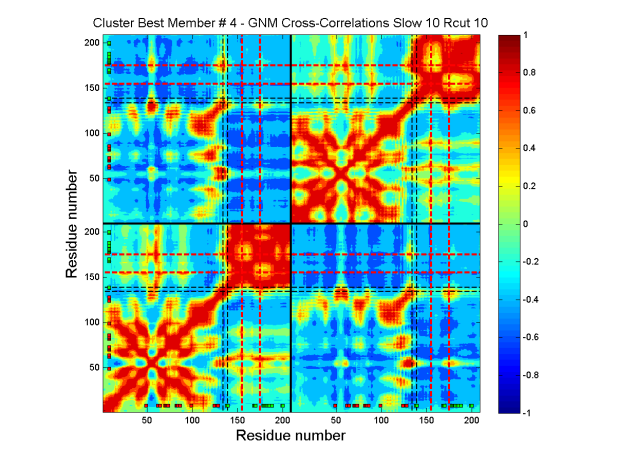  *t = 64 ns* | Cluster #1  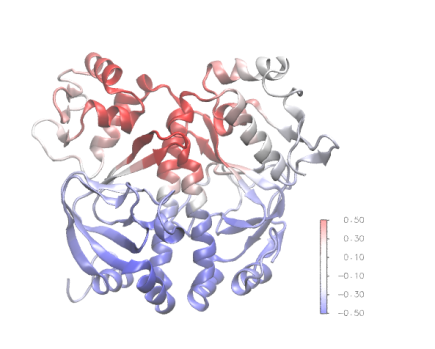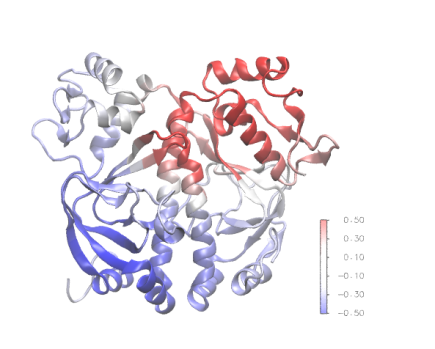 |

| F  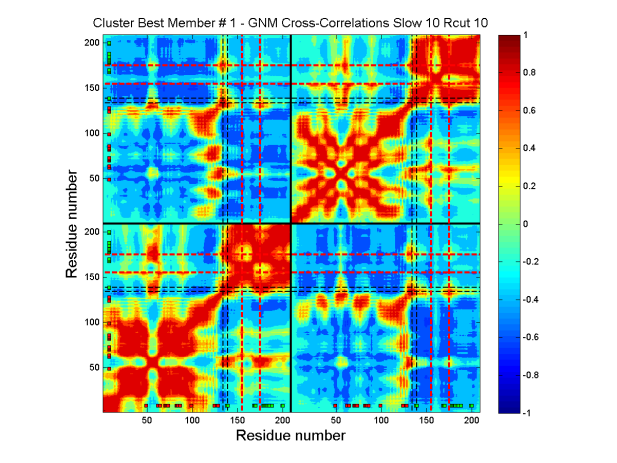  *t = 2 ns* | Cluster #3  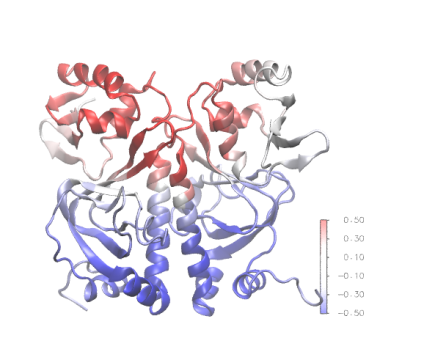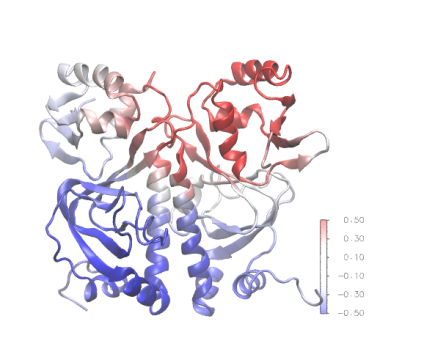 | 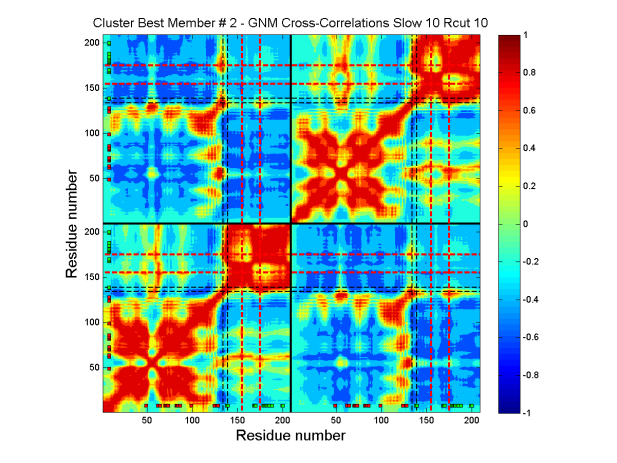  *t = 16 ns* | Cluster #2  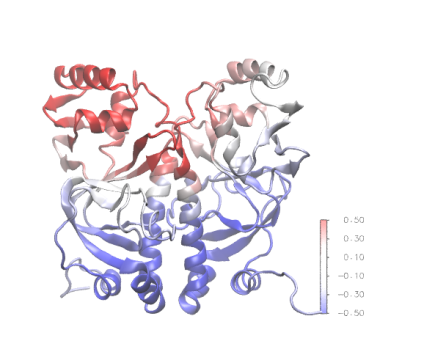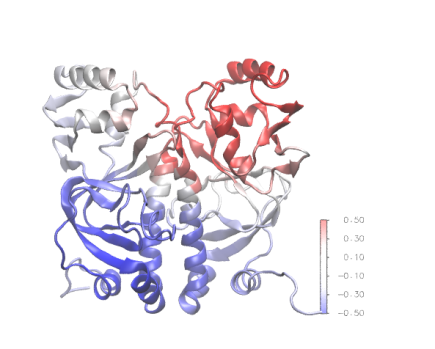 |
| --- | --- | --- | --- |
| 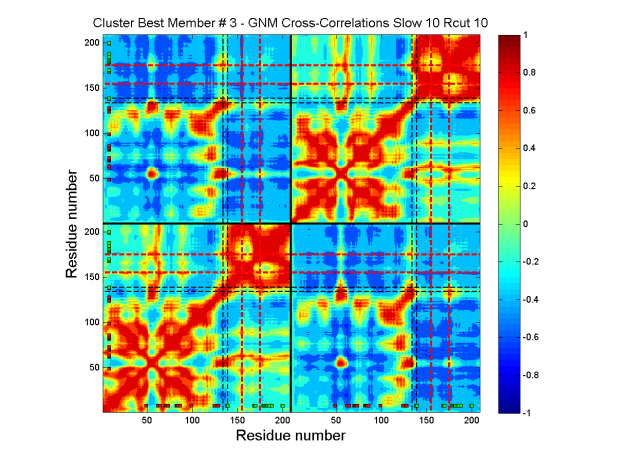  *t = 62 ns* | Cluster #1  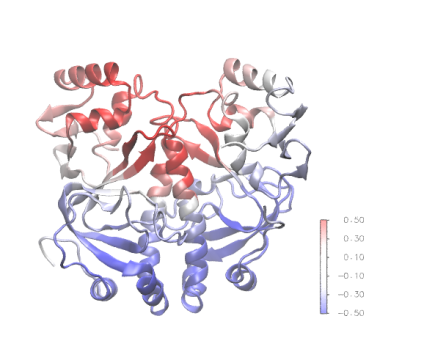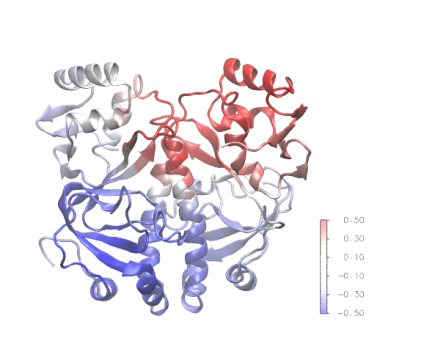 |  |  |

| G  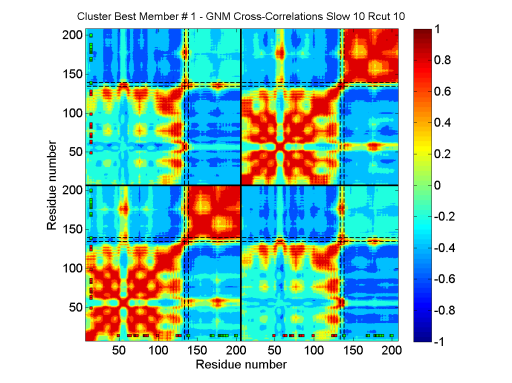  *t = 31.1 ns* | Cluster #1  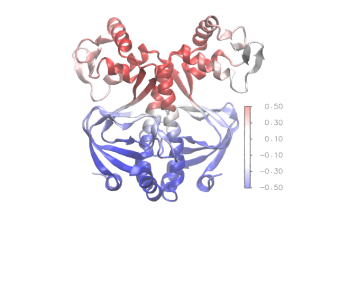  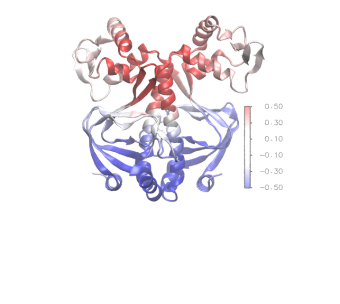 | 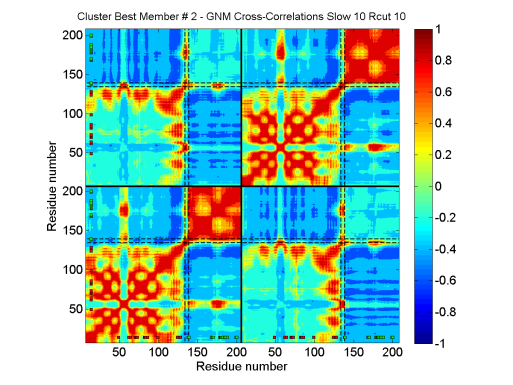  *t = 124.6 ns* | Cluster #2  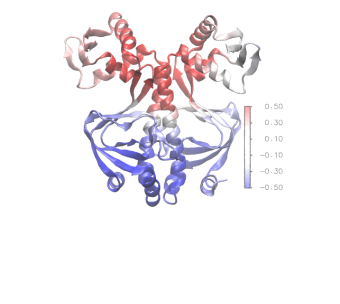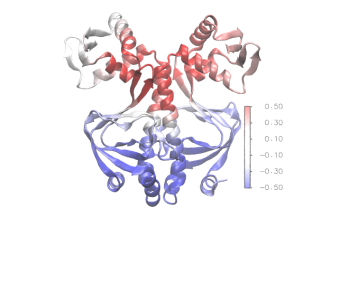 |
| --- | --- | --- | --- |
